# Single-cell RNA-seq transcriptome analysis of linear and circular RNAs in mouse preimplantation embryos

**DOI:** 10.1186/s13059-015-0706-1

**Published:** 2015-07-23

**Authors:** Xiaoying Fan, Xiannian Zhang, Xinglong Wu, Hongshan Guo, Yuqiong Hu, Fuchou Tang, Yanyi Huang

**Affiliations:** Biodynamic Optical Imaging Center (BIOPIC), Peking University, Beijing, 100871 China; College of Life Sciences, Peking University, Beijing, 100871 China; College of Engineering, Peking University, Beijing, 100871 China; Ministry of Education Key Laboratory of Cell Proliferation and Differentiation, Peking University, Beijing, 100871 China; Peking-Tsinghua Center for Life Sciences, Peking University, Beijing, 100871 China; Center for Molecular and Translational Medicine, Peking University Health Science Center, Beijing, 100191 China; Academy for Advanced Interdisciplinary Studies, Peking University, Beijing, 100871 China

## Abstract

Circular RNAs (circRNAs) are a new class of non-polyadenylated non-coding RNAs that may play important roles in many biological processes. Here we develop a single-cell universal poly(A)-independent RNA sequencing (SUPeR-seq) method to sequence both polyadenylated and non-polyadenylated RNAs from individual cells. This method exhibits robust sensitivity, precision and accuracy. We discover 2891 circRNAs and 913 novel linear transcripts in mouse preimplantation embryos and further analyze the abundance of circRNAs along development, the function of enriched genes, and sequence features of circRNAs. Our work is key to deciphering regulation mechanisms of circRNAs during mammalian early embryonic development.

**Electronic supplementary material:**

The online version of this article (doi:10.1186/s13059-015-0706-1) contains supplementary material, which is available to authorized users.

## Background

The transcriptome encompasses all the RNA species transcribed within a cell or an ensemble of cells. Even within the same type of cell, intrinsic heterogeneity exists among the transcriptomes of different individual cells [1]. To fully reveal such complexity, the ideal transcriptome analysis should be performed with individual cells and cover all the RNA species within each cell.

Since we first developed a single cell RNA-seq transcriptome analysis technology in 2009 (the ‘Tang2009’ protocol) [2], a wide variety of single cell RNA-seq methods, such as Smart-seq [3–5], CEL-Seq [6] and Quartz-Seq [7], have been developed. These methods have quickly become powerful tools for dissecting the transcriptome complexity of individual cells, especially in embryonic and neural development, cell reprogramming and cancer progression [4, 8–11].

All of the known single cell RNA-seq protocols for eukaryotic cells are limited to detecting mRNAs with poly(A) tails (poly(A)+ RNAs). There is, however, a substantial amount of non-polyadenylated RNAs (poly(A)- RNAs) expressed in mammalian cells [12]. The standard approach relies on oligo(dT) to prime reverse transcription (RT). Priming through oligo(dT) avoids the preponderance of uninformative ribosomal RNA (rRNA) sequencing reads, which otherwise account for over 90 % of the total RNAs for mammalian cells [13]. However, this approach inevitably precludes the information of other RNA species without the poly(A) tails.

In particular, circular RNAs (circRNAs), a unique set of poly(A)- RNAs [14], have recently been discovered within eukaryotic cells [14–18]. The majority of these circRNAs are formed by exons of coding genes, while some intronic circRNAs were also reported [19, 20]. CircRNAs have been linked to important cellular functions such as the binding and repressing of microRNA (miRNAs) as a sponge [15, 16]. It is desirable to develop a method to detect the *complete* transcriptome, including both poly(A)+ and poly(A)- RNAs, within single cells.

Here we report a novel single-cell transcriptome profiling method, named single-cell universal poly(A)-independent RNA sequencing (SUPeR-seq), using random primers with fixed anchor sequences to replace the commonly used oligo(dT) primers for cDNA synthesis. SUPeR-seq is able to detect both poly(A)+ and poly(A)- RNAs within a single cell with minimal contamination from rRNAs. This method shows higher sensitivity and detects more genes than the Tang2009 protocol. The contamination from genomic DNA and rRNA is negligible. Using SUPeR-seq, we identified in total 141 circRNA transcripts from single HEK293T cells and 2891 circRNA transcripts from single mouse early embryos. In addition, we found hundreds of novel non-circular transcripts by de novo assembly of SUPeR-seq reads generated from individual mouse preimplantation embryos. By comparing the SUPeR-seq reads from mouse oocytes to those from two-cell stage embryos, we identified both maternal and zygotic genes; 81 % of the zygotic genes were further validated by sequencing the two-cell embryos treated with α-Amanitine, a potent inhibitor of gene transcription. These results indicate the high robustness and potential utility of SUPeR-seq.

## Results and discussion

### The sensitivity and accuracy of the SUPeR-seq method

**Fig. 1:**
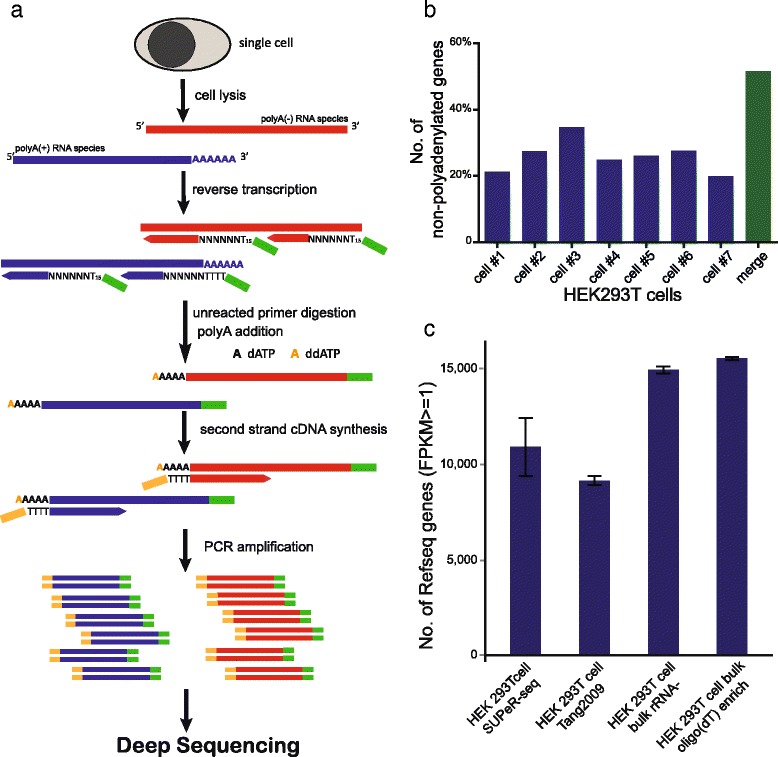
Experimental pipeline of SUPeR-seq, and its sensitivity at the whole-transcriptome scale. **a** The schematic of SUPeR-seq analysis. A single cell is lysed to release RNAs. RNAs are then reverse transcribed into first-strand cDNAs using random primers with a fixed anchor sequence (AnchorX-T_15_N_6_). Unreacted primers are then digested using ExoSAP-IT, followed by adding poly(A) tails to the 3′ ends of the first-strand cDNAs using dATP doped with 1 % ddATP to restrict the length of poly(A) tails. Second-strand cDNAs are synthesized using poly(T) primers with a different anchor sequence (AnchorY-T_24_). Then the double-stranded cDNAs are evenly amplified by PCR using AnchorX-T_15_ and AnchorY-T_24_ primers. Finally the purified single cell cDNAs are used to prepare sequencing libraries following Illumina’s TruSeq DNA sample preparation protocols. **b** Detection sensitivity of SUPeR-seq on poly(A)- genes in individual cells. We identified 696 poly(A)- genes by bulk RNA sequencing, of which around 30 % could be recovered in a single cell by SUPeR-seq (for details, see Additional file 3). When merged the SUPeR-seq data of the seven single cell samples together, over 50 % of these 696 genes could be successfully recovered by SUPeR-seq in at least one cell. **c** The number of genes detected from individual and bulk HEK293T cells using different protocols. SUPeR-seq detected 10,911 genes on average from an individual cell with FPKM ≥1(Fragments Per Kilobase of exon model per Million mapped reads), 19.3 % more than the Tang2009 protocol did (9148 genes on average). For comparison, 14,931 genes were detected with FPKM ≥1 in the four rRNA-depleted total RNA samples from bulk HEK293T cells, and 15,535 genes were detected in the four oligo(dT)-enriched total RNA samples from bulk HEK293T cells

In contrast to our previous Tang2009 protocol that employed oligo(dT)_24_ primers to convert the poly(A)+ mRNAs into cDNAs, SUPeR-seq uses random (AnchorX-T_15_N_6_) primers to enable the simultaneous detection of both poly(A)+ and poly(A)- RNA species from a single cell (Fig. 1a). This primer design also effectively reduced 3′ bias during RT while providing a more balanced sequence coverage along the whole transcript (Fig. S1a in Additional file 1). After the synthesis of the first strand cDNA, we digested the excess primers using ExoSAP-IT to eliminate the formation of primer dimers. Then we added a poly(A) tail to the 3′ end of newly synthesized first-strand cDNA using terminal deoxynucleotidyl transferase (TdT) and dATP doped with 1 % ddATP. The lengths of these artificially added poly(A) tails are crucial because they diminish the sequencing quality if too long whereas they reduce the efficiency of second-strand cDNA synthesis if too short. Using ddATP to terminate the poly(A) extension, we found that a specific ratio of dATP to ddATP (100:1) ensured optimal lengths of poly(A) addition. The second-strand cDNA was subsequently synthesized using a different primer (AnchorY-T_24_) to eliminate primer-dimer formation during the following PCR amplification step. In the second round of PCR, we used 5′-amine-terminated primers to prevent the primers from ligating with Illumina library adaptors, further reducing the amplification bias while improving the sequencing quality.

To determine the coverage of poly(A)- RNA species, we added three types of in vitro transcribed, non-polyadenylated RNAs (green fluorescent protein (GFP), red fluorescent protein (RFP) and Cre RNAs without poly(A) tails; Additional file 2) in each SUPeR-seq reaction of single mouse embryonic stem cells (mESCs). The expression levels of these exogenous spike-ins were linearly correlated with the molecule numbers added, demonstrating that poly(A)- RNAs can be accurately detected (Fig. S1b in Additional file 1). To further confirm the detection of endogenous non-polyadenylated RNAs, we performed three types of transcriptome analyses using total RNA extracted from bulk amounts of HEK293T cells with different enrichment/depletion methods: rRNA-depleted, poly(A)+ mRNA enriched, and co-depletion of rRNA and poly(A)+ RNA. The poly(A)- genes were then identified as the ones that were detected in rRNA-depleted samples at least twofold higher than in poly(A)+ enriched samples (*p* value < 0.05), and also showed high expression levels (FPKM ≥ 1) in the samples with co-depletion of rRNA and poly(A)+ RNA. Using this method, we identified 696 such genes (Additional file 3). SUPeR-seq analysis of a single HEK293T cell covered 30 % of these potential poly(A)- RNAs, and the coverage was over 50 % with the ensemble of seven individual cells (Fig. 1b). We also checked the coverage on histone RNAs [21], and found that SUPeR-seq recovered more poly(A)- RNAs compared with the Tang2009 protocol (Fig. S1c in Additional file 1).

To assess potential genomic DNA contamination, we sequenced oocyte samples both with and without the nuclear region removed. There was no significant difference in the percentage of reads mapped to exons, introns and intergenic regions between them (Fig. S1d in Additional file 1). This indicates that the intron and intergenic regions detected through SUPeR-seq did not result from contamination with genomic DNAs. These non-exon reads might be derived from novel exons of known genes or from primary transcripts before splicing. In addition, the correlation coefficient of gene expression levels between oocyte samples with and without nuclei showed no larger difference compared with that between samples in the same group (Fig. S1e in Additional file 1), indicating that the genomic DNA contamination in the SUPeR-seq data is negligible. Only six genes (*Gm12264*, *Gm12364*, *Gm16832*, *Gm8817*, *Grb14*, *Klrblc*) were detected (FPKM > 1) in these three intact oocytes but absent (FPKM = 0) in the two nuclei-removed oocytes. These genes are likely nuclear-specific transcripts in the mature mouse oocytes. Additionally, we also found three genes (*C87198*, *Atxn7* and *Xlr4a*) with four or more detected reads across the exon–intron boundaries (with at least 10 bp covering both exon and intron regions) only in the intact oocyte samples (but not in the nuclear region removed oocytes). We suspect that such rare pre-splicing primary transcripts were probably due to transcription arrest in MII oocytes [22].

Unexpectedly, SUPeR-seq showed no significant amplification of rRNAs, the major RNA species in a cell. No more than 1.5 % of the total SUPeR-seq reads were mapped to rRNAs (Rn5s, Rn5.8s, Rn18s, and Rn28s) when starting with a single cell or single-cell amount of total RNAs (Fig. S1f in Additional file 1). Several factors might account for the significant preference of SUPeR-seq for mRNAs compared with rRNAs: the limited amount of RNA as starting material, lysis and RT conditions. When the amount of input RNA was increased from 10 pg to 1 ng with SUPeR-seq protocol, the rRNA ratio jumped from 1.2 % to 5.1 % (Fig. S1f in Additional file 1). Also, when we replaced the lysis buffer with commercial RT buffer and followed with the conventional RT step, we indeed observed a higher portion of rRNA reads, but the rRNA mapping ratio was still less than 15 % (Fig. S1f in Additional file 1). Therefore, we speculate that the lysis and RT procedure of SUPeR-seq could not fully break down the strong secondary structures of rRNAs, leading to low RT efficiency of rRNAs.

We further evaluated the sensitivity and accuracy of SUPeR-seq by comparing results by this method with those from multiple bulk samples. We found that SUPeR-seq could detect 10,911 genes (FPKM ≥ 1) within an individual HEK293T cell whereas our previous Tang2009 protocol only detected 9148 genes (FPKM ≥ 1). When compared with rRNA-depleted bulk samples from which 13,773 genes were detected, SUPeR-seq could cover 79 % of these genes from just a single cell (Fig. S2a in Additional file 1). These data show that the SUPeR-seq method can detect the majority of the transcriptome of an individual cell (Fig. 1c). Furthermore, we found that the SUPeR-seq data based on 10 pg, 100 pg and 1 ng total RNA samples all had good concordance with standard RNA-seq of bulk amounts of mESCs (r > 0.85). SUPeR-seq achieved better correlation with regard to gene expression levels with oligo(dT)-enriched bulk RNA-seq than the previous Tang2009 approach did (Fig. S2c in Additional file 1). Also the average Pearson correlation coefficient (r) between gene expression levels in 10-pg total RNA samples and bulk sample was higher than that reported when using Smart-Seq [3]. These results indicate that SUPeR-seq detects gene expression in single cells with higher accuracy than other single cell RNA-seq methods do, and has less systematic bias. The higher consistency with bulk analysis is likely due to the random primers used in both the SUPeR-seq and bulk methods, as they might provide a more uniform coverage on the transcripts. Thus, the SUPeR-seq method is less biased than previous single cell RNA-seq methods.

To evaluate the reproducibility of SUPeR-seq, we performed the experiments using various amounts of pooled RNAs in lieu of single cells to avoid biological variability between samples. A typical mammalian cell contains approximately 10 pg total RNAs. The mean Pearson correlation coefficient (r) for the four technical replicates of 10 pg total RNAs was 0.95, suggesting high reproducibility of SUPeR-seq, comparable to that of Smart-Seq2 [4] (Fig. S2b in Additional file 1). Of the genes with FPKM ≥ 1, 81.7 % show less than a fourfold change between expression levels in two 10 pg replicates of Smart-seq2 [4], while with SUPeR-seq this was slightly lower at 74.3 % (Fig. S2b in Additional file 1) [23]. To address this problem further, we pooled ten HEK293T cells together, lysed them and split them into ten equal fractions to create ten ‘averaged’ single cells, and then processed them separately by SUPeR-seq. We evaluated the technical variations with these ten ‘averaged’ single HEK293T cells. The high correlation among these cells indicates that the quantitative expression levels of the endogenous RNAs are maintained (Fig. S3 in Additional file 1; r = 0.97 on average). By analyzing the coefficient of variance (CV) of the expression levels of the genes in these ‘averaged’ cells, we found that genes with FPKM ≥ 6.3 showed, on average, a mean CV value of less than 1, indicating high precision in quantifying the majority of the expressed genes in these ‘averaged’ cells (Fig. S2d in Additional file 1). For the 696 poly(A)- genes we identified, these cells showed acceptable correlation with each other with an average correlation coefficient of 0.67. Also, the non-poly(A) spike-in molecule RFP could be consistently detected in all ‘average’ single cells (Fig. S2e in Additional file 1), indicating that the relative expression levels of both poly(A)-tailed and non-poly(A)-tailed transcripts could be maintained with SUPeR-seq. When applying the method to mouse oocytes and preimplantation embryos, we also achieved high correlation coefficients between replicates in each stage (Fig. S4a in Additional file 1). Therefore, embryo samples from different stages could be clearly separated according to their gene expression either by principal component analysis or by a multidimensional scaling strategy analysis (Fig. S4b in Additional file 1).

### Analysis of circRNAs in mouse preimplantation embryos by SUPeR-seq

**Fig. 2:**
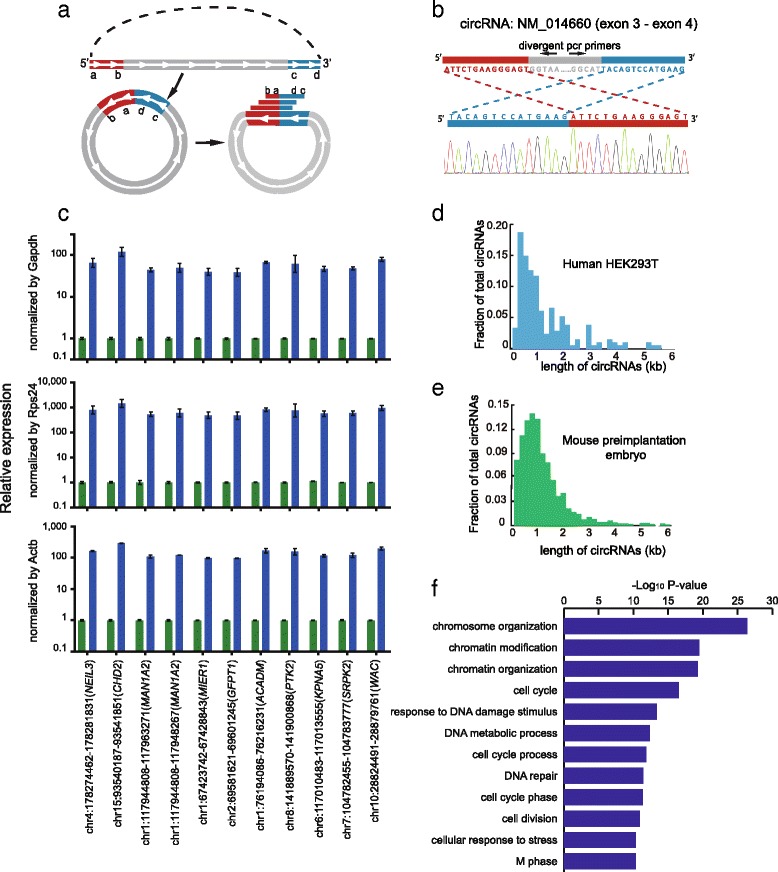
Detection of circRNAs by SUPeR-seq in individual HEK293T cells and mouse preimplantation embryos. **a** Identification of circRNAs in the SUPeR-seq dataset. The termini of two exons are in a sequential order (*a*–-*b*……*c*–-*d*) along the genome, the *red* part shows the upstream exon at the 5′ end of the gene while the *blue* part shows the downstream exon at the 3′ end of the gene (*top*). When looped, the two exons join together from head to tail in a reversed order (*c*–*d*–*a*–*b*, *bottom*). Various sequencing reads covering the junction site can identify the cyclization of a circRNA. **b** Sanger sequencing validation of a newly discovered circRNA through SUPeR-seq in a single HEK293T cell. The end-joining region of the circRNA is PCR amplified to confirm the reversed order of the joined exons. The sequence of the end-joining region is unique to the circRNA but not the host linear RNA. The joint region of the circRNA is chr7:11021999–11030474, and the host gene is *PHF14*. **c** Quantitative RT-PCR of HEK293T cell total RNA treated with RNase R or mock treatment as a control. The circRNA candidates showed ten- to hundred-fold enrichment compared with common linear mRNAs after treatment with RNase R (here we use Gapdh, Rps24 and Actb as linear RNA controls). This clearly demonstrates that all the circRNA candidates are circular. For each circRNA, we made two replicates in the RT-qPCR step. **d** The length distribution of the circRNAs (141 in total) detected in single HEK293T cells. **e** The length distribution of circRNAs (2891 in total) detected in mouse preimplantation embryos. **f** The top GO terms are displayed for 1316 genes from which circRNAs are generated in mouse preimplantation embryos

We then tested if SUPeR-seq was able to detect circRNAs, a unique class of poly(A)- RNA that was recently discovered, and we found abundant circRNAs in mouse preimplantation embryos. Such attempts have been challenging due to limited quantity of starting materials. Hundreds of circRNAs have been previously reported [15] in HEK293T cells through RNA-seq analysis of bulk samples. We developed an analysis pipeline similar to the previous method [15] which can detect junction reads joining the first and last exons of a circRNA (Fig. 2a). By requiring that paired-end reads mapped to the same transcript in a circularized order, we eliminated false positive cases such as trans-splicing or exon tandems. SUPeR-seq of seven single HEK293T cells revealed 141 candidate circRNAs (Additional file 4), out of which we chose 20 for validation; 15 of these were novel and the other five were previously reported. We were able to validate 19 (95 %) of them at single-base resolution by targeted amplification of the end-joining region unique to the circRNAs using RT-coupled PCR (RT-PCR) followed by standard Sanger sequencing (Fig. 2b; Fig. S5 in Additional file 1). These circRNA candidates were also resistant to RNase R treatment, confirming their circularized characteristics (Fig. 2c). Full-length Sanger sequencing of the cDNAs of these circRNA candidates also verified that they were indeed real circRNAs and not linear trans-splicing products between two RNA molecules (Fig. S6 in Additional file 1). Interestingly, we found that the majority of circRNAs are composed of internal exons but not the first and the last exons within the same host gene, and that their lengths are usually shorter than 2 kb (Fig. 2d, e). Most (91 %) circRNAs are formed by multiple exons with only 9 % formed by a single exon. We then applied our method to mouse oocytes and early embryos, including zygotes, two-cell, four-cell, and eight-cell embryos, morulae and blastocysts. circRNAs had never been analyzed at these developmental stages due to the very limited amount of material available for analysis. We identified 2891 circRNAs from 1316 host genes in these early embryo samples (Additional file 4). We selected eight of these circRNAs for independent validation and verified seven of them at single-base resolution in the mouse oocytes by RT-PCR followed by standard Sanger sequencing (Fig. S7 in Additional file 1). This indicates that the majority of novel circRNAs found in mouse preimplantation embryos are authentic circRNAs. We next tested if they had the potential to bind miRNAs as other circRNAs had been reported to function as miRNA sponges in various cell lines [13, 14]. However, only 17 (0.9 %) of these circRNAs contained more than 20 potential miRNA binding sites (Additional file 5), indicating that the majority of the circRNAs in mouse preimplantation embryos are unlikely to play a role as miRNA sponges, which is consistent with previous studies [24]. Gene ontology (GO) analysis of all the 1316 host genes producing these circRNA transcripts showed strong enrichment for terms related to chromatin organization, cell division, and response to DNA damage stimulus (Fig. 2f), suggesting potential roles of these circRNAs in these functional areas.

**Fig. 3:**
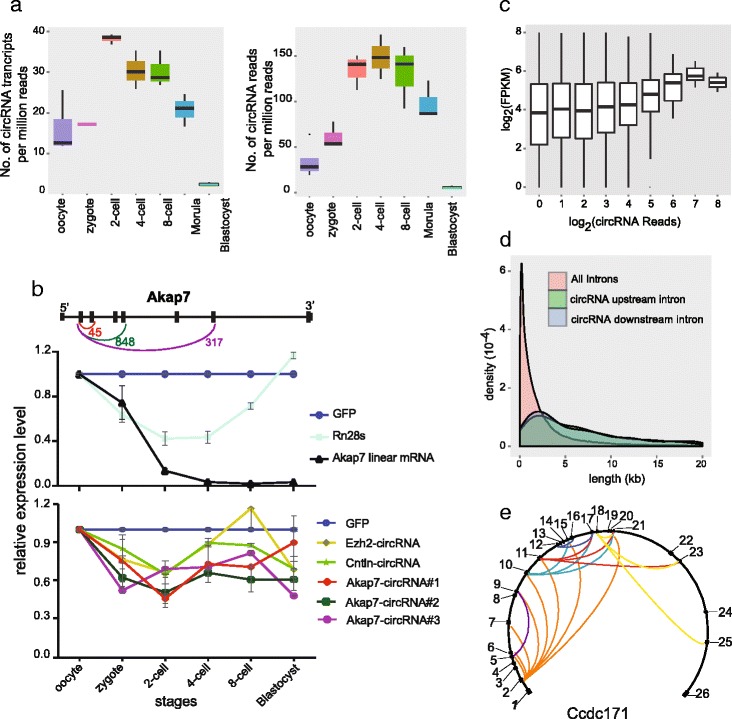
Characterization of the circRNAs expressed in the mouse preimplantation embryos. **a** The number of circRNA transcripts and the number of reads mapping to circRNAs (only counting the reads spanning the end-joining site of circRNAs) per million mapped paired-end reads at each stage of mouse preimplantation embryos. Five biological replicates were used for the oocyte samples, and three biological replicates for each of the other six stages. The concentration of circRNAs is elevated between the two-cell and eight-cell stages and decreased drastically at the morula and blastocyst stages. **b** The expression dynamics of five circRNAs analyzed by RT-qPCR. *Top*: circRNAs generated from the same gene share the 5′ exon to form the head-to-tail junction sites. The arcs show the joint between two exons. The numbers indicate the junction reads for corresponding circRNAs in the SUPeR-seq dataset. CircRNAs with longer downstream introns show higher expression levels as measured by the circRNA-specific end-joining reads. *Middle*: RT-qPCR of the linear transcripts from the mouse oocyte to blastocyst stage. *Bottom*: the abundance of circRNAs analyzed by RT-qPCR from the mouse oocyte to blastocyst stage. GFP RNAs were spiked-in during purification of total RNAs as a control for the technical variation in each sample. *Ezh2*-*circRNA* stands for chr6:47540677–47577667 (exons 2–15 of *Ezh2*), *Cntln*-*circRNA* stands for chr4:84971131–85006524 (exons 6–12 of *Cntln*), *Akap7*-*circRNA*#*1* stands for chr10:25283892–25289730 (exons 2–3 of *Akap7*), *Akap7*-*circRNA*#*2* stands for chr10:25267307–25289730 (exons 2–5 of *Akap7*), *Akap7*-*circRNA*#*3* stands for chr10:25220610–25289730 (exons 2–7 of *Akap7*). The circRNAs are more stable compared with linear ones during the maternal to zygotic transition. **c** The relationship of circRNA read counts with the host gene FPKM. Genes detected with higher circRNA counts also show higher expression levels of linear transcripts. **d** Length distributions of flanking introns of the 2891 circRNAs found in mouse preimplantation embryos. The upstream intron is originally adjacent to the first exon of a circRNA before cyclization and the downstream intron is adjacent to the last exon. Both these specific upstream and downstream introns tend to be much longer than other introns in the same gene. **e** An example (Ccdc171) showing that one host linear RNA gene could generate multiple circRNA species with different joints. All the exons are indicated and numbered along the gene. The arcs between different exons within the circle show end-joining events of the circRNAs

An analysis of the expression dynamics of circRNAs during preimplantation development showed that circRNAs are already expressed in mature oocytes and continue to increase until the four- to eight-cell stage, after which they begin to decline, falling below the oocyte levels by the blastocyst stage (Fig. 3a). Quantitative RT-PCR (RT-qPCR) of several circRNAs during these stages also confirmed the high dynamic nature of circRNA expression (Fig. 3b). The abundance of circRNAs was only about 1 % compared with poly(A)+ RNAs [16]; hence, the circRNA repertoire could hardly be completely discovered in each sample at current sequencing depths. With about 15 million 100-bp paired-end reads sequenced for each sample, it is still far from saturated for coverage of all circRNAs in an individual cell (Fig. S8d in Additional file 1). Notably, in contrast to the global degradation of maternal linear mRNAs during the maternal to zygotic transition of mouse preimplantation embryos, circRNAs seem to be much more stable and resistant to this global degradation process (Fig. 3b).

### Analysis of the formation of the circRNAs in mouse preimplantation embryos

**Fig. 4:**
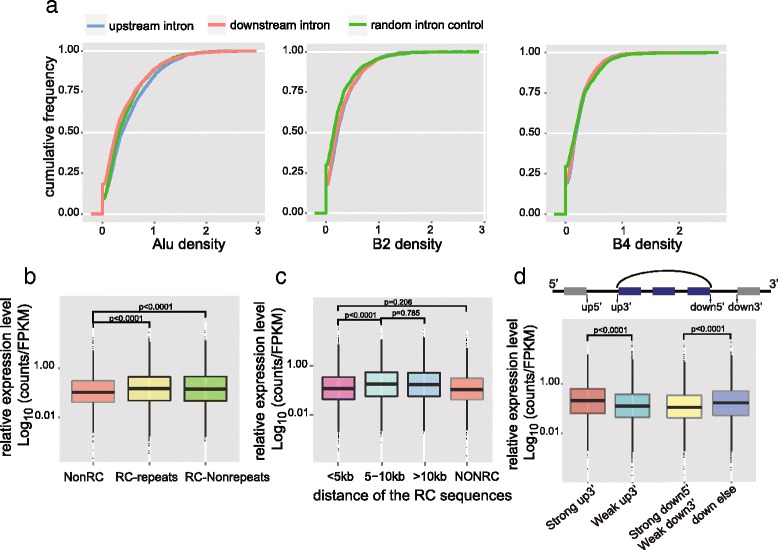
Analysis of reverse complementary sequences and repeat elements for circRNA formation. **a** Three kinds of repeat element density in the introns adjacent to end-joining exons of circRNAs. **b** CircRNAs are divided into three groups according to whether the upstream and downstream introns contain reverse complementary (RC) sequences and whether these sequences belong to repeat elements (*RC*-*repeats* and *RC*-*Nonrepeats*). The circRNA expression levels are normalized by the host gene FPKM. CircRNAs with adjacent introns containing RC sequences shows higher expression than those without RC sequences (*NonRC*). Moreover, the circRNA expression shows positive correlation with the number of RC sequence pairs (Fig. S8c in Additional file 1). **c** We calculated the distance between the nearest pair of RC sequences by summing up their distances to the circRNA splicing sites. When the distance is less than 5 kb, the RC sequences seem not to help circRNA formation. **d** CircRNAs are classed by the strength of the upstream splicing site (*up5*′ and *up3*′) of the first exon forming the end-joining site and those with strong up3′ (*top*) show higher expression than those with weak up3′, while no differences caused by the strength of up5′ were observed. Then when classing the circRNAs by strength of downstream splicing motif (*down5*′ and *down 3*′), only circRNAs with strong down5′ and weak down3′ show higher expression than all the other groups

Several recent studies elucidated the mechanisms of circRNA formation [25, 26], illustrating that circRNAs are not just cellular byproducts [17, 27]. First, we analyzed the characteristics of the circRNAs expressed in the mouse preimplantation embryos. By analyzing the relationship between circRNA amounts and their host gene expression, we found that genes producing larger amounts of circRNAs also showed higher expression of their linear transcripts (Fig. 3c). Second, to help compare the expression levels of different circRNAs, we normalized circRNA expression with their host gene RNA abundance. Then we examined the lengths of the introns of the circRNA host genes and found that the introns flanking the circRNAs before cyclization (median of 7.0 kb and 5.2 kb for the upstream and downstream introns, respectively) were much longer than other introns in the same host gene (1.1 kb on average) or the randomly picked introns (median of 1.2 kb) (Fig. 3d; Fig. S8a in Additional file 1), similar to the situation of longer surrounding introns for circRNAs expressed in H9 cells [25]. Third, it has been shown that one host gene may produce several different circRNAs. This is also consistent with our mouse preimplantation embryo data (Fig. 3b, e). Quite often, circRNAs derived from the same host gene share the same 5′ exon but different 3′ exons for joining (Fig. 3b). Notably, for those circRNA isoforms sharing the same 5′ exon, the ones with longer downstream introns tended to be more abundant (*p* value 1.04e-11). Similarly, for circRNAs sharing the same 3′ exon, the ones with longer upstream introns tended to be more abundant (*p* value 0.03). These trends further support the notion that longer ‘outside’ flanking introns favor the formation of circRNAs (Figs. 3b; Fig. S8b in Additional file 1). Fourth, it has been shown [26, 28–30] that the inversely orientated repeat elements (especially the Alu family) within the introns flanking the circRNAs play important roles in the formation of circRNAs. We further analyzed the repeat elements in the introns flanking the 2891 circRNAs expressed in the mouse preimplantation embryos. The densities of these repeat elements were not significantly different between introns flanking the circRNAs and other random-selected control sequences (Fig. 4a). However, since the introns flanking the circRNAs are much longer, each of them contains, on average, six times more repeats than other introns (Fig. S8a in Additional file 1). This difference indicates that repeat elements might facilitate circRNA formation in mouse preimplantation embryos in vivo, similar to that in cultured cells in vitro. We further classified circRNAs into three groups based on the existence of complementary sequences and repeat elements in adjacent flanking introns, and then compared the normalized expression level (circRNA counts per host gene FPKM) of each group. If the adjacent flanking introns of circRNAs contain complementary sequences, these circRNAs are indeed more abundant. Moreover, the circRNAs with unique non-repeat complementary sequences in their adjacent flanking introns show higher expression levels than those with repeat element complementary sequences in their adjacent flanking introns (Figs. 4b; Fig. S8c in Additional file 1). Interestingly, the complementary sequences in the adjacent flanking introns seem to work only when they are located more than 5 kb away from each other (Fig. 4c). These results agree well with previous findings in cultured cell lines [26, 27], indicating the same mechanism of circRNA formation occurs in both cell lines in vitro and mouse preimplantation embryos in vivo. It was previously shown that linear RNA splicing competed with circRNA formation in the same host gene. When upstream and downstream flanking exons contained splicing sites of stronger motifs, circRNA formation efficiency decreased [25]. We examined if this competition also existed in mouse early embryos. According to our results, circRNAs showed higher relative expression levels when the upstream exons contained stronger splicing motifs, but circRNAs with strong downstream splicing motifs showed less relative expression than circRNAs with weaker downstream splicing motifs (Fig. 4d).

### Analysis of novel linear RNAs in mouse preimplantation embryos by SUPeR-seq

**Fig. 5:**
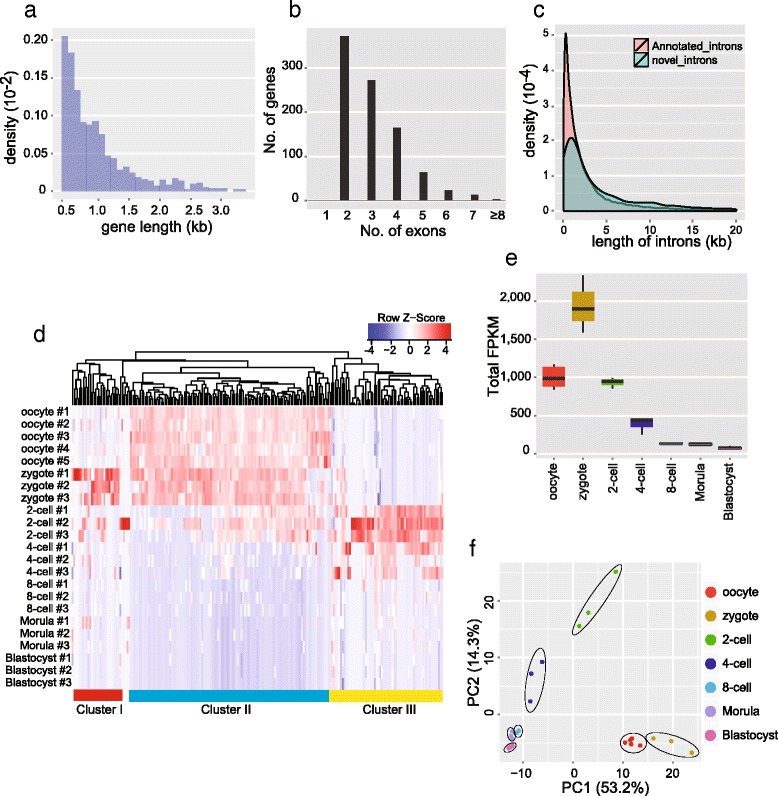
*De novo* assembled novel transcripts in mouse preimplantation embryos. **a** Length distribution for de novo assembled novel transcripts. Candidate novel transcripts with lengths less than 500 bp were filtered out. **b** Exon number distribution for de novo assembled novel transcripts. Candidate novel transcripts with lengths less than 500 bp were filtered out. **c** Intron length distribution for novel candidate transcripts. The introns of novel candidate genes were slightly longer than annotated introns, on average. **d** Hierarchical clustering analysis of novel transcripts, which shows stage-specific expression of genes in mouse preimplantation embryo samples. These genes can be divided into three major clusters: cluster I are early zygotic genes which are expressed during the zygote stage and then are down-regulated at later stages; cluster II are maternal genes, which account for over 60 % of novel genes; cluster III are late zygotic genes which start to be expressed from the two-cell stage. **e** The total FPKM of all 913 novel genes in each embryonic stage, showing enrichment in the embryo stages before the eight-cell stage, especially in zygotes. **f** Principal component (*PC*) analysis of mouse preimplantation embryos based on the 913 novel transcripts

The use of random primers for RT also brings the possibility to identify novel RNAs or genes within mouse preimplantation embryos. Reads mapped to the mouse genome but not to any RefSeq or Ensembl transcripts were extracted, and then de novo assembled using Trinity [31]. The newly assembled transcripts were mapped to the mouse genome with BLAT [32], and only those transcripts that fell into genome regions at least 10 kb away from any annotated Ensembl genes were selected as candidates of novel transcripts. The strand and transcription direction of the novel transcripts were determined by analyzing the intron sequences since the splicing sites usually had strand specificity. Moreover, to exclude any false positive calls from potential genomic DNA contamination, we only considered novel transcripts that contained at least two exons with length over 500 bp and had clear strand specificity. From the SUPeR-seq reads of mouse oocytes, and two-cell, four-cell, eight-cell, morula and blastocyst stage embryos, we assembled 913 novel transcripts in total (Additional file 6). Most of the transcripts (92 %) are separated from other novel transcripts by at least 5 kb in the genome, indicating their independence and uniqueness. These novel transcripts range from 500 bp to 3 kb in length (Fig. 5a, b) and contain longer introns than the annotated transcripts (Fig. 5c). Most of them (95 %) lack coding potential and are probably novel long non-coding RNAs (lncRNAs). We performed hierarchical clustering of the expression levels of these novel transcripts in early embryo samples, and found that most of them showed developmental stage-specific expression patterns. In particular, most novel transcripts are enriched in oocytes and zygotes (Fig. 5d, e). Principal component analysis of these novel genes separated embryos of different developmental stages into distinct groups, indicating that they are potentially functionally relevant for preimplantation development (Fig. 5f), similar to annotated genes which were differentially expressed in each of the embryonic stages (Fig. S4b in Additional file 1).

### Analysis of the maternally and zygotically expressed genes in mouse preimplantation embryos

**Fig. 6:**
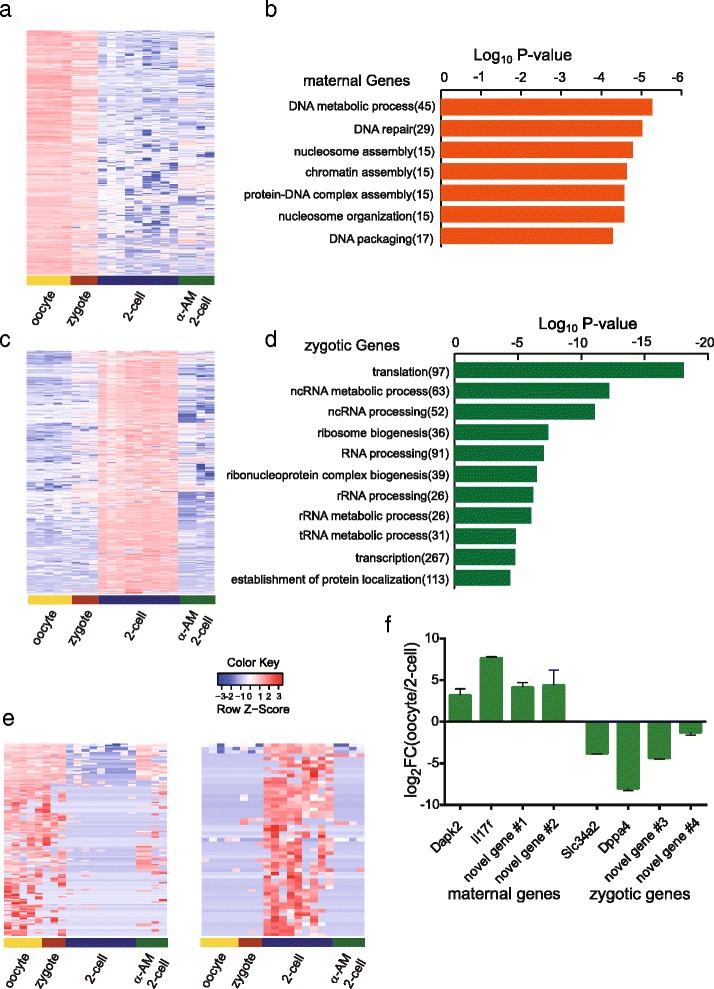
Maternally and zygotically expressed genes in mouse preimplantation embryos. **a** Heatmap of maternally expressed genes. **b** The top enriched GO terms overrepresented in 1238 maternally expressed genes. **c** Heatmap of zygotically expressed genes. The zygotic genes are down-regulated after blocking of transcription with α-Amanitine treatment of the embryos. **d** The top enriched GO terms overrepresented in 4143 zygotically expressed genes. **e** Expression levels of 139 novel maternal genes and 57 novel zygotic genes in mouse oocytes, normal two-cell embryos and α-Amanitine treated two-cell embryos were analyzed. **e** (*lLeft*: the maternal genes show high expression levels in oocyte samples and are largely down-regulated in normal two-cell embryos. Some maternal genes are also still highly expressed in α-Amanitine treated two-cell embryos. (*rRight*: the zygotic genes are highly expressed in normal two-cell embryos. Out of the 57 novel zygotic genes, 54 could be validated, which means they are not transcribed in two-cell embryos treated with 100 ng/ml α-Amanitine. **f** RT-qPCR validation of both annotated and novel maternal and zygotic candidate genes. Maternal genes have ΔΔCt(two-cell-oocyte) > 1 and zygotic genes have ΔΔCt(two-cell-oocyte) < −1. *Novel gene* #*1* to *novel gene* #*4* refer to comp20337_c0, comp46577_c0, comp46858_c0 and comp89562_c0, respectively (also see Additional file 6)

Next we used SUPeR-seq to analyze both the maternal genes and the zygotic genes in mouse embryos. A previous report [33] that relied on microarray analysis was restricted to known genes and had limited sensitivity and a relatively narrow dynamic range. With SUPeR-seq, we identified 1238 maternal genes that were down-regulated from oocytes to two-cell embryos (fold change [Two-cell/oocyte] < 0.25, *p* value < 0.05 Fig. 6a and Additional file 1). GO analysis revealed that these maternally expressed genes were strongly associated with biological terms related to DNA metabolic processes (Fig. 6b). Meanwhile, we also identified 4143 zygotically expressed genes that were up-regulated during zygotic gene activation at the two-cell stage (Fig. 6c; Additional file 7). These zygotic genes are associated with biological terms related to ribosome, translation and transcription process (Fig. 6d). Furthermore, to verify these zygotically expressed genes, we treated mouse zygotes with 100 ng/ml α-Amanitine to globally repress RNA polymerase II dependent transcription and observed that over 81 % (3368 out of 4143) zygotic genes were no longer up-regulated, demonstrating that they were zygotically transcribed (Fig. 6c). Other than these annotated genes, we further identified 139 maternally and 57 zygotically expressed novel transcripts. Fifty-five newly identified zygotic genes were validated by α-Amanitine treatment (Fig. 6e). In addition, we validated four maternal and zygotic genes by RT-qPCR of mouse oocytes and two-cell embryos (Fig. 6f).

### Absolute RNA copy number evaluation

**Fig. 7:**
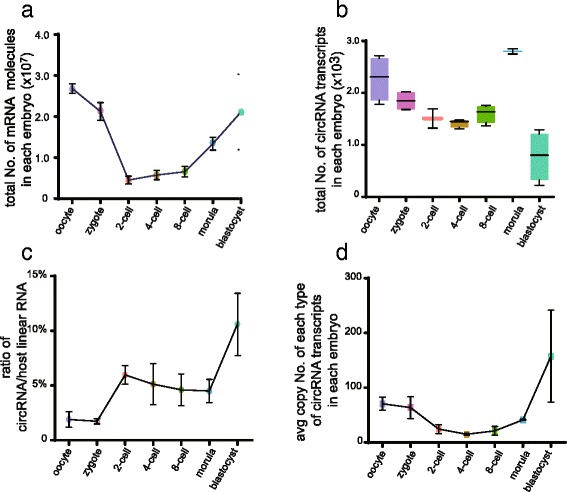
Absolute copy numbers of mRNA molecules in single embryos evaluated by ERCC spike-ins. Absolute copy numbers of mRNAs (**a**) and circRNAs (**b**) in individual embryos at each developmental stage deduced by ERCC spike-ins. **c** The average copy number ratio of circRNAs to their host genes. **d** The average molecule number for each circRNA transcript in every individual embryo for each developmental stage deduced by ERCC spike-ins

It has been shown that the total amount of mRNAs changes drastically during mouse preimplantation development [5]. To analyze the change in absolute RNA copy numbers during mouse preimplantation development, we added External RNA Control Consortium (ERCC) spike-in RNAs into the SUPeR-seq experiments for embryos at each stage. We separated each embryo lysate into two equal halves and ran SUPeR-seq on each. Thus, we could evaluate the technical variations by comparing ERCC spike-in RNAs in the two halves. The copy number of total mRNA molecules fell sharply from 27 million in an oocyte to 4.5 million in a two-cell stage embryo, nearly a sixfold decrease (Fig. 7a and Table 1). Then the total number of mRNA molecules gradually increased after the two-cell stage because of global activation of zygotic genes. When reaching the blastocyst stage, when an embryo consists of 32–64 blastomeres, the total copy number of mRNAs in each embryo was 21 million, comparable to that in an oocyte (Fig. 7a).

**Table 1:** Evaluation of total RNA and circRNA absolute quantity by ERCC spike-in RNAs

Stage	Oocyte	Zygote	2-cell	4-cell	8-cell	Morula	Blastocyst
Average number of mRNA molecules per embryo (×106)	26.8	21.3	4.5	5.7	6.6	13.8	21.1
Average number of circRNA molecules per embryo	2278	1850	1509	1422	1602	2799	779
Ratio of circRNA/host linear RNAR	1.6 %	1.7 %	2.2 %	4.7 %	4.2 %	6.8 %	9.8 %
Average number per type of circRNA transcript per embryo	71	64	24	15	21	41	158
Number of circRNA genes^a^ per stage	363	425	684	517	433	474	123
Number of types of circRNA transcripts per stage	591	718	1,243	861	702	721	140

The total mRNA copy number and circRNA copy number were evaluated through ERCC spike-in

^a^CircRNA host genes; some host genes may generate several different types of circRNA transcripts

We also evaluated the total copy number of circRNA molecules at each embryonic stage through ERCC spike-in RNAs. CircRNAs were relatively abundant in mouse early embryos, with an average of 2278 copies of circRNAs in each mature oocyte, and the number gradually decreased to 1422 copies in a four-cell embryo. Later it increased to 2799 copies in each morula and then dramatically decreased to only 779 copies in each blastocyst (Fig. 7b). This indicates that there is a potentially active degradation process of circRNAs in mouse early embryos between the morula and blastocyst stages.

By comparing the total numbers of circRNA and linear RNA molecules of the host genes in each embryonic stage, we found circRNAs constitute about 10 % of transcripts from the same host genes in the blastocyst stage (Fig. 7c). It seems that a large amount of circRNAs from over 1000 genes were already produced before the mature oocyte stage; then, with the development of the mouse embryo, these circRNAs were gradually degraded, followed by a sharp increase at the morula stage and a sharp decrease at the blastocyst stage (Fig. 7b). However, the average copy number for each type of circRNA transcript greatly increased at the blastocyst stage, indicating that a small number of dominant circRNA transcripts are produced during the global circRNA degradation (Fig. 7d). Considering such dynamic changes of circRNAs during mouse preimplantation development, we speculate that they might play an important role in this process.

## Conclusions

We developed the SUPeR-seq method, a highly sensitive and reproducible method to simultaneously detect both poly(A)+ and poly(A)- RNA species within a single mammalian cell. Using this method, we analyzed a total of 69 mouse mature oocyte and preimplantation embryo samples and detected 2891 circRNAs from 1316 host genes. A majority of these circRNAs are unique to the preimplantation stage and a large proportion of them exhibit dynamic expression patterns during this developmental process. The circRNAs are relatively abundantly expressed in preimplantation embryos, with several thousand copies of circRNAs in each embryo. These circRNAs are potentially involved in chromosome organization, cell cycle regulation, and DNA repair in mouse early embryos. Moreover, these circRNAs show characteristic features, implying their unique splicing patterns, similar to that of circRNAs previously discovered in cultured cell lines. Our work paves the way to decipher functional significance and regulation mechanisms of circRNAs during mammalian early embryonic development.

## Materials and methods

### Animal use and care

All the animal experiments were approved by the Peking University Institutional Animal Care and Use Committee (IACUC) and were performed in compliance with their guidelines.

### mESC total RNA extraction and serial dilution assays

Total RNA was extracted from about one million mESCs or human HEK293T cells with RNeasy Mini Kit (QIAGEN) coupled with on-column DNA digestion following the manufacturer’s protocol. Extracted RNA was quantified with the Qubit® RNA Assay Kit (Invitrogen). The RNA extractions were then diluted to final concentrations of 2 ng/μl, 200 pg/μl and 20 pg/μl. After dilution SUPeR-seq was immediately performed on two replicates of 0.5 μl of each extracted RNA sample to evaluate the technical accuracy and reproducibility of our method.

### Culture of mESCs and human HEK293T cells

mESCs, originally derived from 129S2/Sv mouse [34, 35], were cultured with 2i medium [36] without feeders. HEK293T cells were maintained in Dulbecco's modified Eagle medium (DMEM)/high glucose with 10 % fetal bovine serum, 1× L-glutamine and 1× penicillin-streptomycin. Adherent cells were resuspended with 0.25 % trypsin treatment for 1–3 min. Individual cells were collected with a microcapillary connected to a mouth pipette and washed by transferring them into droplets of 1 mg/ml phosphate-buffered saline-bovine serum albumin for three times before lysis. All cell culture reagents were purchased from Gibco.

### Isolation of mouse oocytes and preimplantation embryos

Around 2-month old CD-1 female mice were superovulated by an injection of 7.5 IU of Pregnant Mare Serum Gonadotropin (PMSG) followed by 7.5 IU (human Chorionic Gonadotrophin) hCG within 46–48 h. MII oocytes were isolated about 17 h after hCG injection and the zona pellucida was removed by treatment with acidic tyrode solution. Then the first polar body was removed from the oocyte before transferring the oocyte into lysis buffer. To analyze the potential genomic DNA contamination in SUPeR-seq, we used Hoechst33342 (Invitrogen) to stain the nuclei of a few MII oocytes and then removed the nuclei by micromanipulation before lysis. Ten hours after the superovulated CD-1 females mated with CD-1 males (assuming the copulation happened at 0:00 am), zygotes were isolated and both the first and second polar bodies were removed by micromanipulation. Two-cell stage embryos were isolated from the oviduct of the mice 36 h after mating. For these embryos, we mechanically dissected the blastomeres of each embryo with a glass needle after removing the zona pellucida and polar bodies. The four-cell, eight-cell and morula stage embryos were isolated from mouse oviduct at 44 h, 57 h and 64 h after copulation. Then some morulae were cultured in KSOM (Millipore) for another 20 h to obtain blastocysts.

### Single cell cDNA amplification

Single cells were lysed to release all RNAs, which then were reverse transcribed into first cDNA strands by SuperScript III reverse transcriptase (Invitrogen) and random primers with an anchor sequence (AnchorX-T_15_N_6_) at 25 °C for 5 min, and 50 °C for 30 min. Then the reverse transcriptase was inactivated by heat treatment at 70 °C for 15 min. We digested the unreacted primers with ExoSAP-IT (USB). Then poly(A) tails were added to the first-strand cDNAs at their 3′ ends by terminal deoxynucleotidyl transferase (Invitrogen) with final dATP and ddATP concentrations of 3 mM and 30 μM, respectively. Next, the second-strand cDNAs were synthesized using poly(T) primers with another anchor sequence (AnchorY-T_24_). These cDNAs were then pre-amplified by PCR with 16 cycles of 95 °C for 30 s, 67 °C for 1 min, 72 °C for 6 min (plus 12 s more after each cycle). Then the PCR products were purified by PCR Purification Kit (QIAGEN) and 0.2–5 kb fragments were recovered from agarose-gel electrophoresis. These purified cDNAs were further amplified by another ten cycles of PCR using poly(T) primers with anchor sequences and C6-amine-blocked 5′ ends (NH_2_-AnchorX-T_15_ and NH_2_-AnchorY-T_24_). After purification, the cDNAs were ready for sequencing library preparation. The sequence of AnchorX is 5′-GTCGACGGCGCGCCGGATCCATA-3′ and the sequence of AnchorY is 5′-ATATCTCGAGGGCGCGCCGGATCC-3′.

### Sequencing library generation and next-generation sequencing of single cell cDNA samples

Typically 200–300 ng amplified cDNAs were sonicated to around 200 bp long fragments using a Covaris acoustic shearing instrument and then used to construct Illumina sequencing libraries following Illumina’s TruSeq DNA sample preparation protocols. The fragmented cDNAs were end-repaired, a single A base was added to the 3′ end, and then ligated with illumina PE adaptors. Then the ligated DNA fragments were enriched and amplified with ten cycles of PCR. The libraries were sequenced on either Illumina’s HiSeq 2000, or HiSeq 2500 instruments with 100-bp pair-end reads. All clusters that passed the quality filter were exported into fastq files.

### Sequencing library generation for bulk amount of mouse ES cell RNA and HEK293T cell RNA

Total RNA (1 μg) was used for deep-sequencing library construction following the instructions of the TruSeq RNA sample preparation kit (Illumina). The final quality-ensured libraries were sequenced on a HiSeq 2000 sequencer generating 100 bp paired-end reads.

### Single cell lysis buffer replacement

The original lysis condition of SUPeR-seq consists of 0.9× PCR buffer II and 5 mM MgCl_2_ (Geneamp), 0.45 % NP40 (Roche), 4.5 mM DTT (Invitrogen), 0.36 U/ul RNase inhibitor (Invitrogen), 0.18 U/μl SUPERase-In (Invitrogen), 0.125 mM dNTP (Takara) and 50 nM RT primer in a total volume of 4.5 μl. When checking whether the bias on rRNA is because of the cell lysis components, we replaced the lysis buffer with conventional RT buffer which consisted of 1× First Strand Buffer (Invitrogen), 4.5 mM DTT (Invitrogen), 0.36 U/μl RNase inhibitor (Invitrogen), 0.25 mM dNTP (Takara) and 50 nM RT primer in a total volume of 4.5 μl. In both conditions, the lysis reaction set as 70 °C for 90 s on the thermocycler.

### rRNA-depleted HEK293T cell total RNA library preparation

Total RNA (5 μg) was used for removing the rRNAs using Ribo-Zero rRNA Removal Kits (Epicentre) following the manufacturer's instructions. The rRNA-depleted RNAs were then used for library construction using the Illumina TruSeq RNA sample preparation kit.

### Alignment of sequencing reads and gene-expression analysis

The raw reads were filtered with a quality control pipeline in Perl script to remove low quality reads (reads with 50 % of bases with quality value ≤ 5 and >10 % bases undetermined). The adaptor sequences and poly (A)_24_/(T)_24_ sequences were trimmed off. We also removed the reads with AT content higher than 80 %, which were probably a byproduct of cDNA synthesis. We mapped the filtered reads to mm10/hg19 with TopHat (version 2.0.6) [37]. Then gene expression levels were calculated and normalized as FPKM with Cufflinks (version 2.1.1) [38]. The gene annotation GTF files for mm10 and hg19 were downloaded from Ensemble (release 73) and Gencode (v18), respectively.

### Poly(A)- gene detection in HET293T cells

We prepared four replicates of rRNA-depleted RNA-seq samples, four replicates of oligo(dT)-enriched RNA-seq samples and three replicates depleted of both rRNA and poly(A)-tailed mRNA samples. The expression level (FPKM) of GENCODE genes was estimated using Cufflinks (version 2.1.1). Student’s t-test was used to calculate the *p* value between expression of genes from two groups. Genes that showed at least a twofold enrichment in the rRNA-depleted group compared with the oligo(dT)-enriched group (*p* value < 0.05), and at the same time with FPKM > 1 in the group in which both rRNA and polyadenylated RNA were depleted, were considered as poly(A)- genes. Also, genes with length less than 300 bp were filtered out.

### Detection of circRNAs from single cells

For every filtered read with length over 60 bp, anchor-pair sequences were extracted by cutting the first and last 25 bp of the read. Two fastq files were generated, with the first storing all anchors in the first 25 bp and the second storing the last 25 bp anchor sequences in order. Then anchor pairs were mapped to the mm10/hg19 genome with bowtie2. The anchor pairs flanking the joining end of a circRNA should be mapped to the same chromatin within 100 kb at the same strand but in the reversed order. To improve reliability, we only considered circRNA events which were joined by two exons from a single Ensembl transcript. We further inspected the pair-mate of the anchor pairs, and for most of the circRNAs, the pair-mate was mapped to the same gene and within the span of the circRNAs.

To verify our data analysis pipeline, we processed the raw data from Memczak et al. [16]. In our pipeline, we mainly considered the circRNAs with junctions using the well-annotated splicing sites. We found 191 circRNAs, out of which 173 matched with those reported by Memczak et al., an overlap ratio of 93.5 %. On the other hand, we adopted Memczak’s analysis pipeline to process our experimental data. For SUPeR-seq data of seven single HEK293T cells, the Memczak pipeline and our pipeline reported 119 and 141 circRNAs with at least two junction reads, respectively, and the overlap between them was 113 (overlap coverage ratio of 86.9 %). These results clearly demonstrated the sensitivity and reliability of our data analysis pipeline. The pipeline presented by Memczak et al. [16] did report circRNAs with unannotated splicing sites when analyzing our SUPeR-seq data. However, we randomly chose five of them (chr11:118363842–118372449, chr1:178745855–178846761, chr1:182567912–182571214, chr22:30093929–30094454, chr5:78533474–78611974) to validate the junction sites joining the first and last exons of the circRNA by RT-PCR and all failed to generate a band with the expected size when running the PCR products on agarose gels.

### circRNA validation by RT-PCR

Twenty circRNAs from the circRNA list of HEK293T cells and eight circRNAs from the list of mouse oocytes were picked out for verification. For each circRNA we designed a pair of divergent PCR primers at the circRNA position on the genome. Then the HEK293T cell or the mouse oocyte total RNAs reverse transcribed with random primers were used as a PCR template. PCR amplification was carried out by the following program: 95 °C for 5min followed by 35 cycles of 95 °C for 30 s, 54 °C for 30 s and 72 °C for 30 s, then 72 °C for 7 min. The PCR products were then run on 1.5 % agarose gels. The predicted strands were cut out directly for Sanger sequencing.

### Maternal and zygotic gene validation and RT-qPCR of circRNAs to detect expression along mouse preimplantation embryos

For each stage, about 40 embryos were isolated and total RNA was extracted with the RNeasy Micro Kit (QIAGEN). When lysing the embryos in RLT buffer, corresponding GFP molecules were added (for each embryo we added 10^6^ GFP molecules). The RNAs were eluted in 10 μl elution buffer and reverse transcribed by SuperScript III (Invitrogen). The cDNAs in the reaction buffer were precipitated by 2.5 volumes of alcohol. The cDNAs in the sediments were resuspended by nuclease-free water according to the initial number of embryos (1 μl H_2_O for two embryos). Then qPCR was carried out using these dissolved cDNAs. For circRNAs we used the divergent primers on the genome to amplify the end-joining site. For the linear RNAs, we designed primers on the last exon of the genes.

### RNase R resistance validation of circRNA candidates

HEK293T cell total RNA (4 μg) was treated with RNase R (Epicentre) or nuclease-free water (mock control) at 37 °C for 15 min. Then the treated RNAs were reverse transcribed with random primers by SuperScript III (Invitrogen). The cDNAs were then used as qPCR templates.

### Analysis of the sensitivity and reproducibility of SUPeR-seq

The sequencing data from four 10-pg mESC total RNA samples were used to analyze the technical reproducibility of SUPeR-seq. We calculated the pairwise Pearson correlation coefficients of FPKM values between samples. Genes that did not show any expression in both samples were excluded from the calculation. The gene expression of two 10-pg samples by Smart-seq2 were downloaded from the Gene Expression Omnibus database (accession GSE49321) [4]. The sequencing data from different methods were processed and gene expression level was estimated in the same way using TopHat (version 2.0.6) and Cufflinks (version 2.2.1). The gene annotation GTF file was downloaded from GENCODE (version V18).

### De novo assembly of new transcripts and genes

For mouse preimplantation embryonic cells, we de novo assembled RNA reads that did not map to annotated genes. Together there were 29 early embryonic samples including five oocytes, three zygotes, six two-cell stage blastomeres, and three embryos each for the two-cell, four-cell, eight-cell, morula and blastocyst stages. Reads unmapped to RefSeq and Ensembl were exported to a fastq format file and then transferred to a Trinity [37] pipeline. After filtering the transcripts with length less than 500 bp, the remaining transcripts were mapped to genome genes with BLAT [38], and the structure of the novel transcripts was recovered including introns and exons. To exclude possible genome contamination and increase the accuracy of prediction, we only considered the transcripts containing at least two exons. To exclude the possibility of relationship with known Ensembl genes, we removed the transcripts that had Ensembl genes in the neighboring 10 kb up- or downstream along the genome. Furthermore, we predicted the strand of the transcripts based on the notion that the first and last two bases of almost all introns is GT and AG. The GTF files of these 913 genes were output and the expression levels of the assembled transcripts (FPKM) were estimated with Cufflinks (version 2.1.1).

### Analysis of maternal and zygotic genes

The number of fragments that mapped to each mouse Ensembl gene was counted with Cufflinks (version 2.2.1) in all five oocyte, nine two-cell stage and four α-Amanitine-treated two-cell stage embryonic samples. Then we used the edgeR package in R to detect the differentially expressed genes. The maternal genes were defined as those that showed fourfold enrichment in oocytes and a *p* value < 0.05, while zygotic genes showed a fourfold enrichment in two-cell stage embryos. We identified 4143 annotated zygotic genes and 1238 annotated maternal genes under the criteria above. At the same time, we identified 139 novel maternal genes and 57 novel zygotic genes. Global validation of zygotic genes was performed by comparing gene expression levels in normal two-cell embryos to those in 100 ng/ml α-Amanitine-treated two-cell embryos: 3368 out of 4143 (81 %) annotated zygotic genes and 55 out of 57 novel zygotic genes (96 %) showed fourfold down-regulation in α-Amanitine treated samples.

### Reverse complementary sequences between introns upstream and downstream of circRNAs

Blastn was used to analyze the reverse complementary sequences between the flanking introns of circRNAs with the upstream intron sequence being the subject and downstream intron sequence being the query. The parameter is word_size 11 -gapopen 5 -gapextend 2 -penalty −3 -reward 2. Reverse complementary sequences longer than 25 were considered.

### Data processing workflow for circRNA analysis

1. Raw data from illumina Hiseq2000 or Hiseq2500
2. Quality control (QC): cut adaptor and low quality reads
3. Map to genome: using TopHat2 default setting
4. Output unmapped reads: using Samtools view -f 4
5. Create anchor reads: cut 25 bp from two ends of each 100 bp read
6. Map anchor reads to genome: using Bowtie2 default setting
7. Filter candidate reads: find sequence with two anchors mapping to the same chromsome of opposite directions within distance <200 kb
8. Filtering with existing exon junctions: the two anchors of candidate circRNA reads are then mapped to the exons within the same transcript and they must match the flanking sequences of exons.

### Accession codes

Raw sequencing data, mapped data and data for visualization of all SUPeR-seq analyses have been deposited to the Gene Expression Omnibus (accession GSE53386).

## Additional files

Additional file 1:
**Maternal and zygotic genes found in the mouse embryos.** **Figure S1**. SUPeR-seq could detect non-poly(A) genes without rRNA or genome contamination. **Figure S2**. SUPeR-seq shows high sensitivity, reproducibility and more accuracy. **Figure S3**. Correlations of gene expression levels among the pool-and-split HEK293T cells. **Figure S4**. SUPeR-seq achieves high correlation between biological replicates. Figure S5. Validation of circRNAs in HEK293T cells. **Figure S6**. CircRNA full-length validation. **Figure S7**. CircRNA validation in mouse oocytes. CircRNA abundance is related to introns adjacent to exons forming the circRNA. **Figure S8**. CircRNA abundance is related to introns adjacent to exons forming the circRNA. Additional file 2:
**Spike-in RNAs in SUPeR-seq mESC samples.**
Additional file 3:
**Poly(A)- genes in HEK293T cells.**
Additional file 4:
**CircRNAs in mouse preimplantation embryos and HEK293T cells.**
Additional file 5:
**MicroRNA binding sites on the circRNAs.**
Additional file 6:
**De novo assembled genes in the mouse preimplantation embryos.**
Additional file 7:
**Maternal and zygotic genes found in the mouse embryos.**

## Abbreviations

bp: base pair
circRNA: circular RNA
ERCC: External RNA Control Consortium
GFP: green fluorescent protein
GO: gene ontology
kb: kilobase
mESC: mouse embryonic stem cell
miRNA: microRNA
rRNA: ribosomal RNA
FPKM: Fragments Per Kilobase of exon model per Million mapped reads
PCR: polymerase chain reaction
RFP: red fluorescent protein
RT: reverse transcription
RT-qPCR: quantitative RT-PCR
SUPeR-seq: single cell universal poly(A)-independent RNA sequencing
