# Supplementary material for: Single-cell RNA-seq transcriptome analysis of linear and circular RNAs in mouse preimplantation embryos
Source: Genome Biol. 2015 Jul 23;16(1):148. doi: 10.1186/s13059-015-0706-1 (PMC4511241; doi:10.1186/s13059-015-0706-1)
Supplement: Additional file 1: — Maternal and zygotic genes found in the mouse embryos. Figure S1. SUPeR-seq could detect non-poly(A) genes without rRNA or genome contamination. Figure S2. SUPeR-seq shows high sensitivity, reproducibility and more accuracy. Figure S3. Correlations of gene expression levels among the pool-and-split HEK293T cells. Figure S4. SUPeR-seq achieves high correlation between biological replicates. Figure S5. Validation of circRNAs in HEK293T cells. Figure S6. CircRNA full-length validation. Figure S7. CircRNA validation in mouse oocytes. CircRNA abundance is related to introns adjacent to exons forming the circRNA. Figure S8. CircRNA abundance is related to introns adjacent to exons forming the circRNA. [file 13059_2015_706_MOESM1_ESM.zip › Sup.F1 SUPeR-Seq detect nonpolyA genes without rRNA or genomic contamination.pdf]

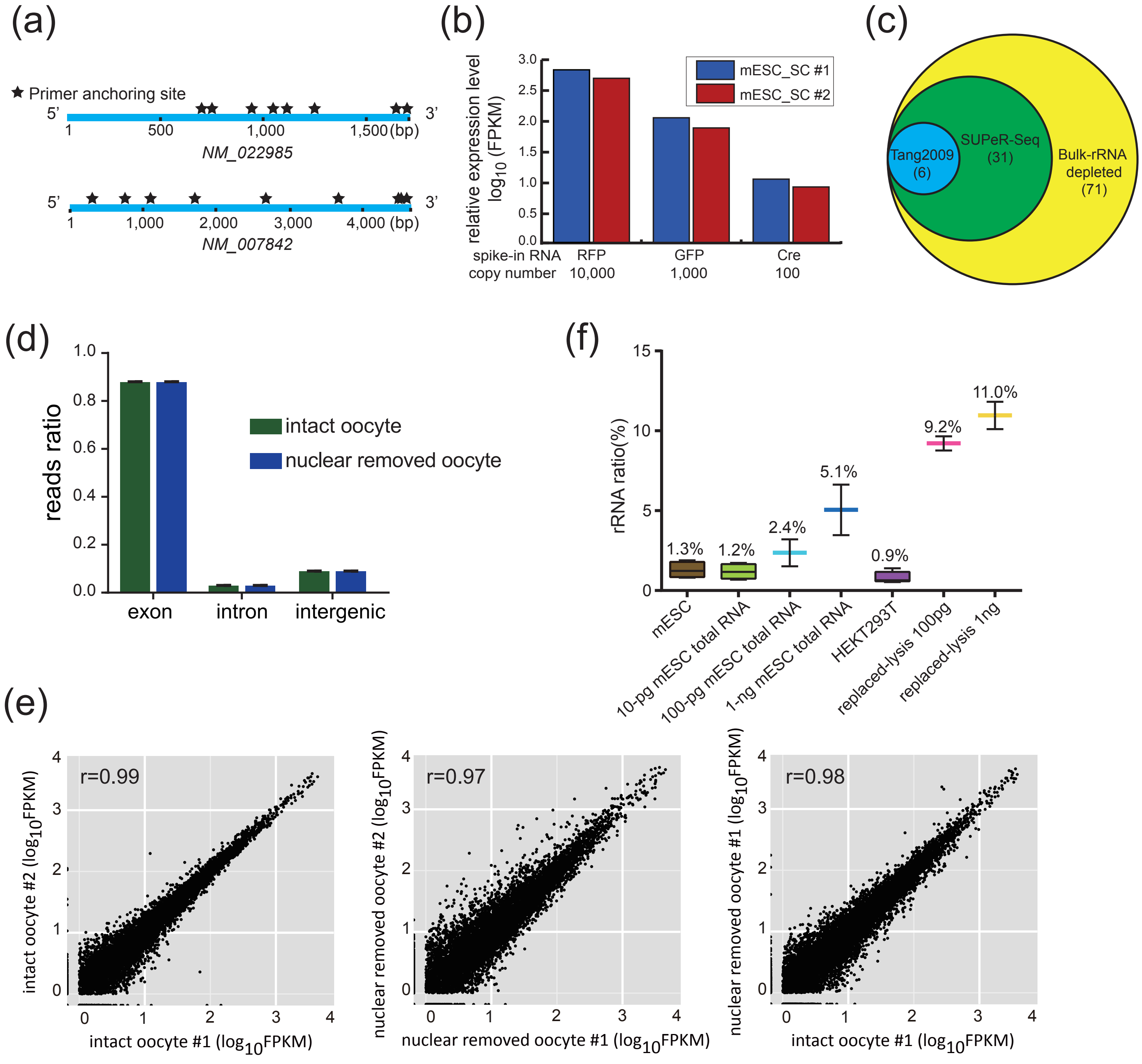

Figure s1. SUPeR-seq could detect non-poly(A) genes without rRNA or genome contamination
(a) The RT primers with N_6 hexmer could attach onto the inner sequences as well as poly(A) sequence in 3'UTR. Two examples: NM_022985 with total length of 1,658bp and NM_007842 with total length of 4,623bp. The blue bars stands for the transcripts and the numbers below shows the lengh location. The black stars shows the detected RT starting site along the transcripts. (b) Expression of three kinds of in vitro transcribed RNA spike-ins (RFP, GFP, and Cre) without poly(A) tails at their 3' ends. (c) The coverage of three Tang2009 and seven SUPeR-Seq single cell samples on histone RNAs, as well as four rRNA-depleted bulk samples. (d) reads ratio are calculated by the number of reads mapped to exon, intron and intergenic region normalizing to the number of total mapped reads in the five oocyte samples. (e) scater plot of two intact oocyte samples (left), two nuclear region removed oocyte samples (middle), and an intact oocyte and a nuclei removed oocyte (right). (f) rRNA ratio of high throughput sequencing reads were calculated, samples including four single m-ESCs, four 10-pg mESC total RNA, two100-pg mESC total RNA,two1-ng mESC total RNA , seven single HEK-293T cells and mouse ESC total RNA samples treated with replaced cell lysis buffer.
