# Supplementary material for: Single-cell RNA-seq transcriptome analysis of linear and circular RNAs in mouse preimplantation embryos
Source: Genome Biol. 2015 Jul 23;16(1):148. doi: 10.1186/s13059-015-0706-1 (PMC4511241; doi:10.1186/s13059-015-0706-1)
Supplement: Additional file 1: — Maternal and zygotic genes found in the mouse embryos. Figure S1. SUPeR-seq could detect non-poly(A) genes without rRNA or genome contamination. Figure S2. SUPeR-seq shows high sensitivity, reproducibility and more accuracy. Figure S3. Correlations of gene expression levels among the pool-and-split HEK293T cells. Figure S4. SUPeR-seq achieves high correlation between biological replicates. Figure S5. Validation of circRNAs in HEK293T cells. Figure S6. CircRNA full-length validation. Figure S7. CircRNA validation in mouse oocytes. CircRNA abundance is related to introns adjacent to exons forming the circRNA. Figure S8. CircRNA abundance is related to introns adjacent to exons forming the circRNA. [file 13059_2015_706_MOESM1_ESM.zip › Sup.F2 SUPeR-Seq shows high sensitivity and reproducibility.pdf]

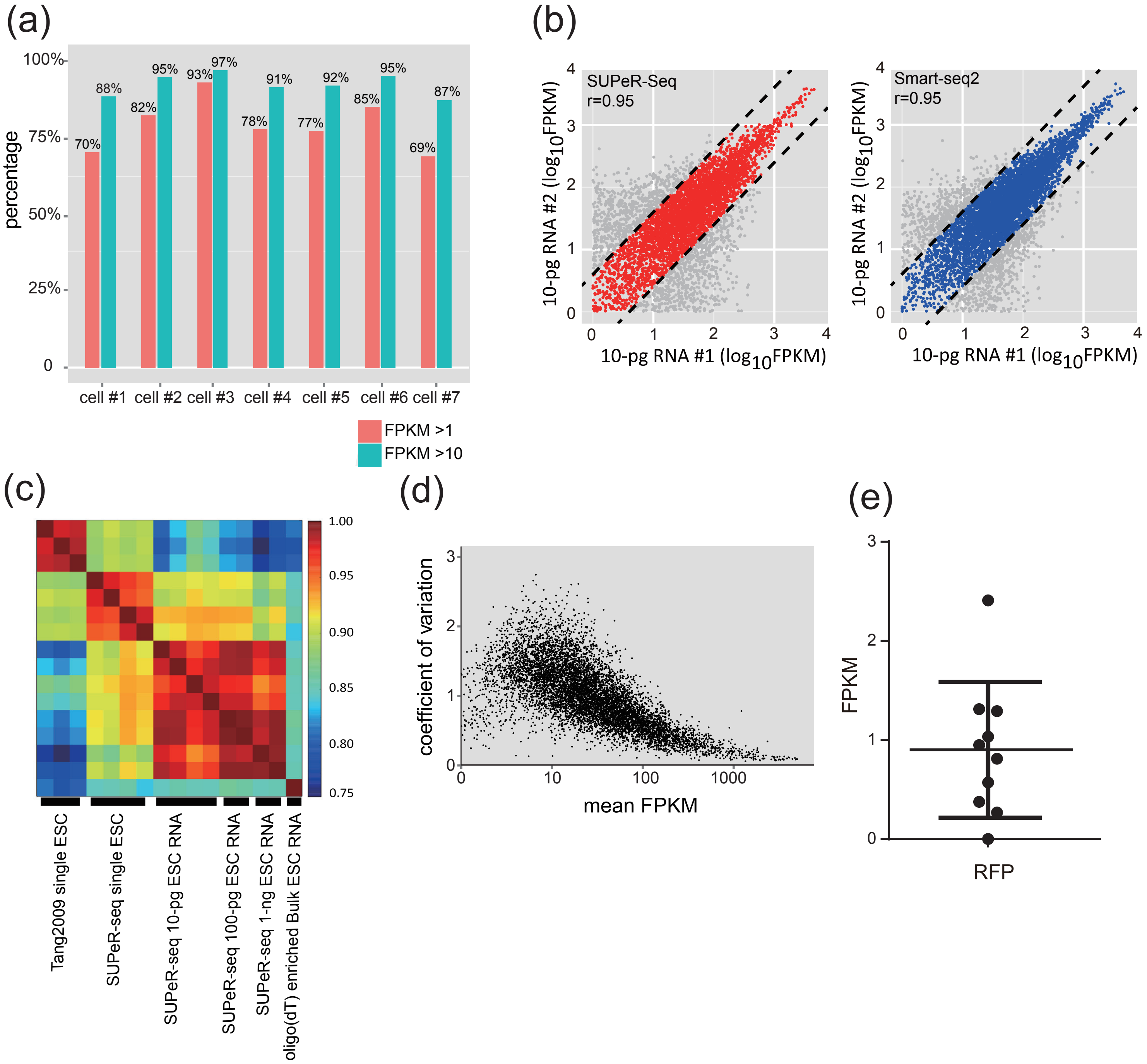

Figure s2. SUPeR-seq shows high sensitivity, reproducibility and more accuracy

(a) The Ensembl genes with FPKM ≥1 and FPKM ≥10 in the four rRNA-depleted samples are 13,773 and 7,058, respectively. SUPeR-seq of single HEK293T cells have an average coverage of 79% and 92% on these genes. (b) Scatter diagram of two 10-pg mESC total RNA replicates with SUPeR-seq and two 10-pg HEK293T cell total RNA replicates with Smart-seq2, respectively. The dash line reflects the four fold changes of gene expression between each two replicates. 74.3% (red dots) and 81.7% (blue dots) genes are detected within four fold changes by SUPeR-seq and Smart-seq2, respectively. The Smart-seq2 data was downloaded from GSE49321. (c) The technical accuracy and reproducibility of SUPeR-seq was assessed through serial dilution of total RNAs extracted from mESCs (1 ng, 100 pg and 10 pg), all these replicates shows high correlation (r>0.95). SUPeR-seq shows less systematic error than Tang2009 protocol when comparing to oligo(dT) enriched bulk RNA-seq data. (d) Coefficient variance are ploted against the mean expression levels for all genes detected with FPKM ≥0.1 in ten pool-and-split HEK293T cells. (e)The expression level of the spike-in non-poly(A) RNA molecule RFP in the 10 pool-and-split HEK293T cell samples.
