# Supplementary material for: Single-cell RNA-seq transcriptome analysis of linear and circular RNAs in mouse preimplantation embryos
Source: Genome Biol. 2015 Jul 23;16(1):148. doi: 10.1186/s13059-015-0706-1 (PMC4511241; doi:10.1186/s13059-015-0706-1)
Supplement: Additional file 1: — Maternal and zygotic genes found in the mouse embryos. Figure S1. SUPeR-seq could detect non-poly(A) genes without rRNA or genome contamination. Figure S2. SUPeR-seq shows high sensitivity, reproducibility and more accuracy. Figure S3. Correlations of gene expression levels among the pool-and-split HEK293T cells. Figure S4. SUPeR-seq achieves high correlation between biological replicates. Figure S5. Validation of circRNAs in HEK293T cells. Figure S6. CircRNA full-length validation. Figure S7. CircRNA validation in mouse oocytes. CircRNA abundance is related to introns adjacent to exons forming the circRNA. Figure S8. CircRNA abundance is related to introns adjacent to exons forming the circRNA. [file 13059_2015_706_MOESM1_ESM.zip › Sup.F6 circRNA full length.pdf]

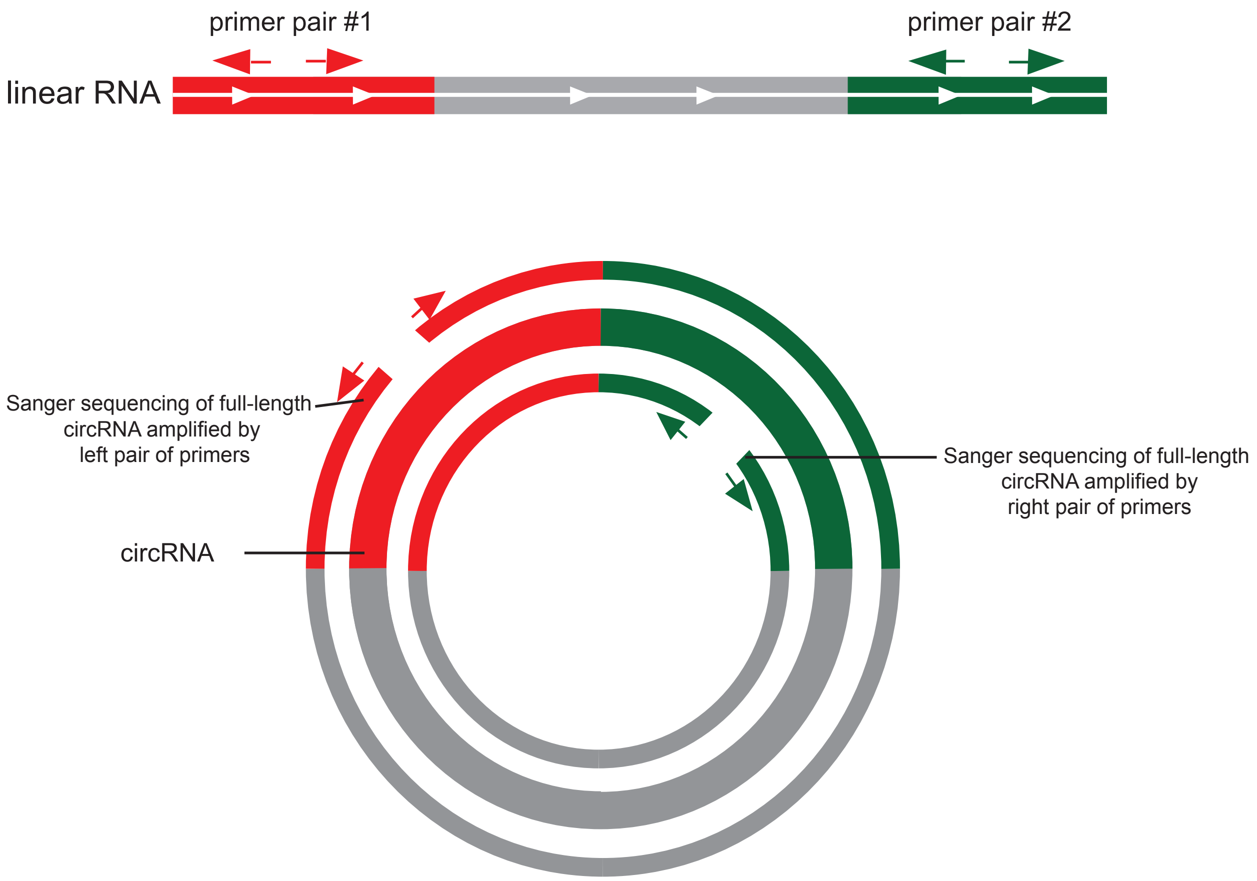

PCR product amplified by primer pair #1:
CAAGAGGAAAAACCCTCTGAAGATCAAATCCACAAGCTGTTACCAGAGGATACAGAAACAGGGAAAAGGAAAAT
GGATGAACAGAAAAAAAGAGATGAACCATTAGTACTGAAAACAAATCTGGAACGTTGTCCTGCACGTCTCTCAGA
TTCAGAGAATGAAGAACCTTCTCGAGGCCAGATGACACAGACACATCGCTCGGCATTTGTTTCCAAGAACAACTC
CTACTCCTTAGCTTTCCTGGCAGGACTTTATATTCAGAGCACCAATCAAATTAAGCAAGCCTGGGG
PCR product amplified by primer pair #2:
CTCGGCATTTGTTTCCAAGAACAACTCCTACTCCTTAGCTTTCCTGGCAGGACTTTATATTCAGAGCACCAATCAA
ATTAAGCAAGCCTGGGGAACTTCGTGAGGAATATGAAAGCTTGAGAAAGCTGAGAGAAGAAAAGTTACAAGAGG
AAAAACCCTCTGAAGATCAAATCCACAAGCTGTTACCAGAGGATACAGAAACAGGGAAAAGGAAAATGGATGAAC
AGAAAAAAAGAGATGAACCATTAGTACTGAAAACAAATCTGGAACGTTGTCCTGCACGTCTCTCAGATTCAGAGA
ATGAAGAACCTTCTCGAGG

Figure s6. CircRNA full-length validation
To verify the circRNA candidates consisted of all the exons in between the exons constructing the end-joining cyclization region (red and green orthogons), divergent PCR primers were designed on each exon region (red and green arrows), then the PCR products were further validated by sanger sequencing. PCR products from both sides of primers could crossover the cyclization point and cover the exons sequences as speculated. The sequences showed above was just an example of circRNA: chr11:74500671-74528759, which contains exons 2-4 of NM_001098638.
