# Supplementary material for: Single-cell RNA-seq transcriptome analysis of linear and circular RNAs in mouse preimplantation embryos
Source: Genome Biol. 2015 Jul 23;16(1):148. doi: 10.1186/s13059-015-0706-1 (PMC4511241; doi:10.1186/s13059-015-0706-1)
Supplement: Additional file 1: — Maternal and zygotic genes found in the mouse embryos. Figure S1. SUPeR-seq could detect non-poly(A) genes without rRNA or genome contamination. Figure S2. SUPeR-seq shows high sensitivity, reproducibility and more accuracy. Figure S3. Correlations of gene expression levels among the pool-and-split HEK293T cells. Figure S4. SUPeR-seq achieves high correlation between biological replicates. Figure S5. Validation of circRNAs in HEK293T cells. Figure S6. CircRNA full-length validation. Figure S7. CircRNA validation in mouse oocytes. CircRNA abundance is related to introns adjacent to exons forming the circRNA. Figure S8. CircRNA abundance is related to introns adjacent to exons forming the circRNA. [file 13059_2015_706_MOESM1_ESM.zip › Sup.F8 intron length of circRNA.pdf]

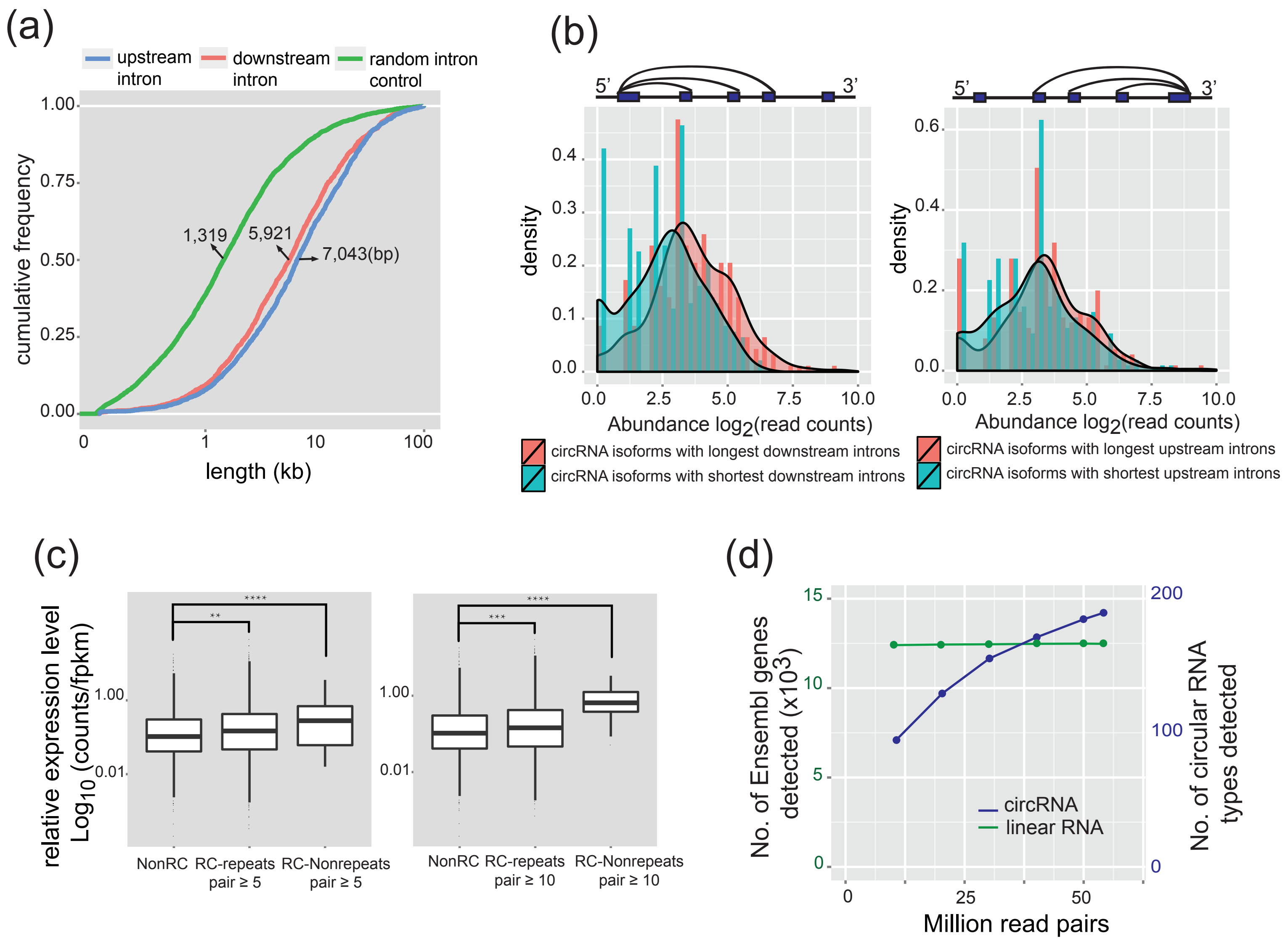

Figure s8. circRNA abundance is related to introns adjacent to exons forming the circRNA. (a) The intron lengths are about 6 times longer than random chosen introns in the genome. (b) For circRNA isoforms sharing the same 5'(3') exons but different 3'(5') exons (as shown in the top of the diagram), we plotted the distribution of the abundance of isoforms with the longest downstream(upstream) introns (colored in red), as well as the abundance of isoforms with the shortest downstream(upstream) introns (colored in blue). The bar/density plot shows that the isoforms with the longer introns in the downstream(upstream) tend to obtain higher abundance, indicating probably easier for circulization. (c) circRNA expression shows positive correlation to number of the RC sequence pairs in the introns adjacent to exons forming the circRNAs. Also see Fig. 4b. (d) In order to check the robustness of SUPeR-seq on circRNAs, an oocyte sample was deep sequenced to over 50 million read pairs (100bp x 2). The circRNA number detected (blue) keeps increasing with deeper sequencing depth while the linear RNA number (green) already saturated with less than 10 million read pairs.
