# Supplementary figures and images for: Single-cell RNA-seq transcriptome analysis of linear and circular RNAs in mouse preimplantation embryos
Source: Genome Biol. 2015 Jul 23;16(1):148. doi: 10.1186/s13059-015-0706-1 (PMC4511241; doi:10.1186/s13059-015-0706-1)

(a)

| oocyte |      |      |      |      | zygote |      |      | 2-cell |      |      | 4-cell |      |      | 8-cell |      |      | morula |      |      | blastocyst |      |      |
|--------|------|------|------|------|--------|------|------|--------|------|------|--------|------|------|--------|------|------|--------|------|------|------------|------|------|
| 1.00   | 0.99 | 0.98 | 0.97 | 0.93 | 0.73   | 0.81 | 0.76 | 0.44   | 0.61 | 0.50 | 0.22   | 0.18 | 0.20 | 0.08   | 0.10 | 0.09 | 0.12   | 0.13 | 0.13 | 0.12       | 0.10 | 0.10 |
| 0.99   | 1.00 | 0.98 | 0.95 | 0.91 | 0.72   | 0.79 | 0.74 | 0.42   | 0.57 | 0.47 | 0.23   | 0.20 | 0.22 | 0.09   | 0.10 | 0.09 | 0.13   | 0.13 | 0.14 | 0.11       | 0.10 | 0.09 |
| 0.98   | 0.98 | 1.00 | 0.96 | 0.94 | 0.74   | 0.81 | 0.76 | 0.42   | 0.58 | 0.46 | 0.21   | 0.17 | 0.19 | 0.08   | 0.09 | 0.09 | 0.11   | 0.12 | 0.12 | 0.10       | 0.10 | 0.09 |
| 0.97   | 0.95 | 0.96 | 1.00 | 0.95 | 0.78   | 0.83 | 0.79 | 0.50   | 0.68 | 0.52 | 0.26   | 0.22 | 0.24 | 0.10   | 0.11 | 0.11 | 0.14   | 0.15 | 0.15 | 0.14       | 0.12 | 0.11 |
| 0.93   | 0.91 | 0.94 | 0.95 | 1.00 | 0.76   | 0.82 | 0.77 | 0.41   | 0.59 | 0.43 | 0.23   | 0.19 | 0.21 | 0.10   | 0.10 | 0.11 | 0.13   | 0.14 | 0.14 | 0.13       | 0.12 | 0.11 |
| 0.73   | 0.72 | 0.74 | 0.78 | 0.76 | 1.00   | 0.97 | 0.98 | 0.53   | 0.68 | 0.51 | 0.26   | 0.22 | 0.25 | 0.10   | 0.10 | 0.11 | 0.13   | 0.15 | 0.14 | 0.14       | 0.13 | 0.11 |
| 0.81   | 0.79 | 0.81 | 0.83 | 0.82 | 0.97   | 1.00 | 0.98 | 0.48   | 0.66 | 0.49 | 0.23   | 0.19 | 0.22 | 0.08   | 0.09 | 0.09 | 0.11   | 0.12 | 0.12 | 0.12       | 0.11 | 0.10 |
| 0.76   | 0.74 | 0.76 | 0.79 | 0.77 | 0.98   | 0.98 | 1.00 | 0.50   | 0.68 | 0.51 | 0.23   | 0.19 | 0.22 | 0.08   | 0.08 | 0.08 | 0.10   | 0.13 | 0.12 | 0.11       | 0.11 | 0.09 |
| 0.44   | 0.42 | 0.42 | 0.50 | 0.41 | 0.53   | 0.48 | 0.50 | 1.00   | 0.88 | 0.96 | 0.77   | 0.68 | 0.75 | 0.42   | 0.47 | 0.47 | 0.47   | 0.54 | 0.52 | 0.40       | 0.35 | 0.38 |
| 0.61   | 0.57 | 0.58 | 0.68 | 0.59 | 0.68   | 0.66 | 0.68 | 0.88   | 1.00 | 0.88 | 0.49   | 0.41 | 0.48 | 0.23   | 0.25 | 0.25 | 0.27   | 0.34 | 0.33 | 0.28       | 0.21 | 0.25 |
| 0.50   | 0.47 | 0.46 | 0.52 | 0.43 | 0.51   | 0.49 | 0.51 | 0.96   | 0.88 | 1.00 | 0.76   | 0.65 | 0.76 | 0.40   | 0.45 | 0.44 | 0.44   | 0.48 | 0.44 | 0.38       | 0.34 | 0.37 |
| 0.22   | 0.23 | 0.21 | 0.26 | 0.23 | 0.26   | 0.23 | 0.23 | 0.77   | 0.49 | 0.76 | 1.00   | 0.93 | 0.97 | 0.68   | 0.71 | 0.73 | 0.63   | 0.63 | 0.65 | 0.47       | 0.48 | 0.47 |
| 0.18   | 0.20 | 0.17 | 0.22 | 0.19 | 0.22   | 0.19 | 0.19 | 0.68   | 0.41 | 0.65 | 0.93   | 1.00 | 0.93 | 0.83   | 0.84 | 0.86 | 0.78   | 0.77 | 0.79 | 0.61       | 0.62 | 0.62 |
| 0.20   | 0.22 | 0.19 | 0.24 | 0.21 | 0.25   | 0.22 | 0.22 | 0.75   | 0.48 | 0.76 | 0.97   | 0.93 | 1.00 | 0.71   | 0.76 | 0.76 | 0.68   | 0.66 | 0.70 | 0.53       | 0.51 | 0.50 |
| 0.08   | 0.09 | 0.08 | 0.10 | 0.10 | 0.10   | 0.08 | 0.08 | 0.42   | 0.23 | 0.40 | 0.68   | 0.83 | 0.71 | 1.00   | 0.96 | 0.97 | 0.94   | 0.91 | 0.93 | 0.83       | 0.83 | 0.82 |
| 0.10   | 0.10 | 0.09 | 0.11 | 0.10 | 0.10   | 0.09 | 0.08 | 0.47   | 0.25 | 0.45 | 0.71   | 0.84 | 0.76 | 0.96   | 1.00 | 0.96 | 0.94   | 0.92 | 0.94 | 0.80       | 0.78 | 0.79 |
| 0.09   | 0.09 | 0.09 | 0.11 | 0.11 | 0.11   | 0.09 | 0.08 | 0.47   | 0.25 | 0.44 | 0.73   | 0.86 | 0.76 | 0.97   | 0.96 | 1.00 | 0.94   | 0.92 | 0.93 | 0.79       | 0.80 | 0.79 |
| 0.12   | 0.13 | 0.11 | 0.14 | 0.13 | 0.13   | 0.11 | 0.10 | 0.47   | 0.27 | 0.44 | 0.63   | 0.78 | 0.68 | 0.94   | 0.94 | 0.94 | 1.00   | 0.96 | 0.97 | 0.82       | 0.80 | 0.80 |
| 0.13   | 0.13 | 0.12 | 0.15 | 0.14 | 0.15   | 0.12 | 0.13 | 0.54   | 0.34 | 0.48 | 0.63   | 0.77 | 0.66 | 0.91   | 0.92 | 0.92 | 0.96   | 1.00 | 0.98 | 0.80       | 0.80 | 0.80 |
| 0.13   | 0.14 | 0.12 | 0.15 | 0.14 | 0.14   | 0.12 | 0.12 | 0.52   | 0.33 | 0.44 | 0.65   | 0.79 | 0.70 | 0.93   | 0.94 | 0.93 | 0.97   | 0.98 | 1.00 | 0.80       | 0.80 | 0.80 |
| 0.12   | 0.11 | 0.10 | 0.14 | 0.13 | 0.14   | 0.12 | 0.11 | 0.40   | 0.28 | 0.38 | 0.47   | 0.61 | 0.53 | 0.83   | 0.80 | 0.79 | 0.82   | 0.80 | 0.80 | 1.00       | 0.98 | 0.98 |
| 0.10   | 0.10 | 0.10 | 0.12 | 0.12 | 0.13   | 0.11 | 0.11 | 0.35   | 0.21 | 0.34 | 0.48   | 0.62 | 0.51 | 0.83   | 0.78 | 0.80 | 0.80   | 0.80 | 0.80 | 0.98       | 1.00 | 0.97 |
| 0.10   | 0.09 | 0.09 | 0.11 | 0.11 | 0.11   | 0.10 | 0.09 | 0.38   | 0.25 | 0.37 | 0.47   | 0.62 | 0.50 | 0.82   | 0.79 | 0.79 | 0.80   | 0.80 | 0.80 | 0.98       | 0.97 | 1.00 |

(b)

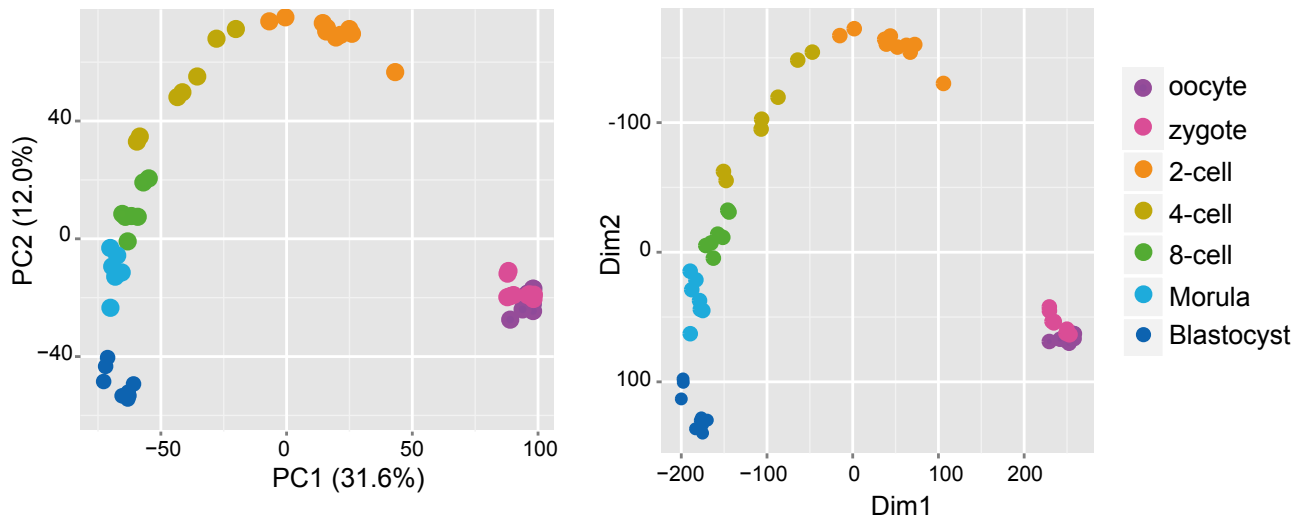

Supplement: Additional file 1: — Maternal and zygotic genes found in the mouse embryos. Figure S1. SUPeR-seq could detect non-poly(A) genes without rRNA or genome contamination. Figure S2. SUPeR-seq shows high sensitivity, reproducibility and more accuracy. Figure S3. Correlations of gene expression levels among the pool-and-split HEK293T cells. Figure S4. SUPeR-seq achieves high correlation between biological replicates. Figure S5. Validation of circRNAs in HEK293T cells. Figure S6. CircRNA full-length validation. Figure S7. CircRNA validation in mouse oocytes. CircRNA abundance is related to introns adjacent to exons forming the circRNA. Figure S8. CircRNA abundance is related to introns adjacent to exons forming the circRNA. [file 13059_2015_706_MOESM1_ESM.zip › Sup.F4 correlation of embryos.pdf]
