# Supplementary material for: Single-cell RNA-seq transcriptome analysis of linear and circular RNAs in mouse preimplantation embryos
Source: Genome Biol. 2015 Jul 23;16(1):148. doi: 10.1186/s13059-015-0706-1 (PMC4511241; doi:10.1186/s13059-015-0706-1)
Supplement: Additional file 2: — Spike-in RNAs in SUPeR-seq mESC samples. [file 13059_2015_706_MOESM2_ESM.pdf]

Additional file 2. Spike-in RNAs in SUPeR-seq mouse ESC samples

RNA	Sequence (5’ - 3’)
GFP	GCTTTACTTGTACAGCTCGTCCATGCCGAGAGTGATCCCGGCGGCGGTCACGAACTCCAGCAG<br>GACCATGTGATCGCGCTTCTCGTTGGGGTCTTTGCTCAGGGCGGACTGGGTGCTCAGGTAGTG<br>GTTGTCGGGCAGCAGCACGGGGCCGTCGCCGATGGGGGTGTTCTGCTGGTAGTGGTCGGCG<br>AGCTGCACGCTGCCGTCCTCGATGTTGTGGCGGATCTTGAAGTTCACCTTGATGCCGTTCTTCT<br>GCTTGTCGGCCATGATATAGACGTTGTGGCTGTTGTAGTTGTACTCCAGCTTGTGCCCCAGGAT<br>GTTGCCGTCCTCCTTGAAGTCGATGCCCTTCAGCTCGATGCGGTTCACCAGGGTGTCGCCCTCG<br>AACTTCACCTCGGCGCGGGTCTTGTAGTTGCCGTCGTCCTTGAAGAAGATGGTGCGCTCCTGG<br>ACGTAGCCTTCGGGCATGGCGGACTTGAAGAAGTCGTGCTGCTTCATGTGGTCGGGGTAGCG<br>GCTGAAGCACTGCACGCCGTAGGTCAGGGTGGTCACGAGGGTGGGCCAGGGCACGGGCAG<br>CTTGCCGGTGGTGCAGATGAACTTCAGGGTCAGCTTGCCGTAGGTGGCATCGCCCTCGCCCTC<br>GCCGGACACGCTGAACTTGTGGCCGTTTACGTCGCCGTCCAGCTCGACCAGGATGGGCACCA<br>CCCCGGTGAACAGCTCCTCGCCCTTGCTCACCATGGTGGCGACCGGCGATGGATCCTGCAAAA<br>AGAACAAGTAGCTTGTATTCTATAGTGT
RFP	CCTTGGAGCCGTACTGGAACTGAGGGGACAGGATGTCCCAGGCGAAGGGCAGGGGGCCGCC<br>CTTGGTCACCTTCAGCTTGGCGGTCTGGGTGCCCTCGTAGGGGCGGCCCTCGCCCTCGCCCTC<br>GATCTCGAACTCGTGGCCGTTCACGGAGCCCTCCATGCGCACCTTGAAGCGCATGAACTCCTT<br>GATGACGTCCTCGGAGGAGGCCATGGTGGCGGGATCCTGCAAAAAGAACAAGTAGCTTGTAT<br>TCTATAGTGT
Cre	CGCCGCATAACCAGTGAAACAGCATTGCTGTCACTTGGTCGTGGCAGCCCGGACCGACGATGA<br>AGCATGTTTAGCTGGCCCAAATGTTGCTGGATAGTTTTTACTGCCAGACCGCGCGCCTGAAGAT<br>ATAGAAGATAATCGCGAACATCTTCAGGTTCTGCGGGAAACCATTTCCGGTTATTCAACTTGCA<br>CCATGCCGCCCACGACCGGCAAACGGACAGAAGCATTTTCCAGGTATGCTCAGAAAACGCCTG<br>GCGATCCCTGAACATGTCCATCAGGTTCTTGCGAACCTCATCACTCGTTGCATCGACCGGTAAT<br>GCAGGCAAATTTTGGTGTACGGTCAGTAAATTGGACACCTTCCTCTTCTTCTTGGGCATAGATC<br>CTGCAAAAAGAACAAGTAGCTTGTATTCTATAGTGT
